# Impact of Biodeterioration on the Structural and Thermal Insulation Properties of Plant Fibre-Reinforced Polymer Biocomposites for Construction Applications

**DOI:** 10.3390/ma19153353

**Published:** 2026-08-06

**Authors:** Elżbieta Stanaszek-Tomal

**Affiliations:** Faculty of Civil Engineering, PK Cracow University of Technology, 24 Warszawska Street, 31-155 Cracow, Poland; estanaszek-tomal@pk.edu.pl

**Keywords:** natural fibre composites, biodeterioration, moisture transport, fungal degradation, hygrothermal ageing, construction materials, durability, polymer biocomposites

## Abstract

**Highlights:**

**What are the main findings?**

- Moisture is the main trigger linking water uptake, fungal activity and damage at the fibre–matrix interphase.
- Moisture and biodeterioration jointly affect functional performance: retained moisture increases thermal conductivity, whereas biological degradation may contribute to persistent microstructural damage and mechanical deterioration.
- ISO 846:2019-type tests are useful for screening, but they do not fully represent porous natural-fibre biocomposites exposed to cyclic building conditions.
- Durability assessment is more reliable when hygrothermal exposure, residual performance, microstructure and biological activity are evaluated together.

**What are the implications of the main findings?**

- The most promising protection strategies combine fibre modification, hydrophobic barriers and antimicrobial or photocatalytic components.
- Future testing should connect biological resistance with thermal and structural performance rather than treating them as separate properties.
- Harmonised protocols for construction biocomposites are needed to improve comparability between studies.
- Predictive approaches that combine diagnostics with numerical and AI-supported modelling may improve service-life estimation.

**Abstract:**

Natural fibre-reinforced polymer biocomposites are increasingly considered for construction applications because of their renewable reinforcement, low density and potentially reduced environmental impact. Their durability, however, is strongly constrained by moisture, which promotes fibre swelling, weakens the fibre–matrix interphase and creates conditions favourable to fungal colonisation. This review examines the effects of moisture and biodeterioration on the structural and thermal performance of natural fibre-reinforced polymer composites used in construction. Across the reviewed studies, moisture and hygrothermal ageing produced tensile-strength reductions ranging from less than 10% to approximately 40%, while losses in stiffness or impact performance exceeded 40–70% in particularly susceptible systems. In contrast, directly comparable quantitative data linking fungal biodeterioration with changes in thermal conductivity remain scarce, representing a major research gap. Current durability standards, particularly ISO 846:2019, provide useful screening data but do not fully reproduce the cyclic hygrothermal conditions experienced by porous, hygroscopic construction biocomposites. More reliable durability assessment requires integrated microbiological, hygrothermal, mechanical and microstructural testing supported by advanced non-destructive diagnostics. Protection strategies should combine moisture control and biological resistance while considering long-term service performance, environmental impacts, recyclability and end-of-life constraints.

## 1. The Role of Biocomposites in Sustainable Construction

Reducing the environmental impact of construction is now a central problem in materials engineering. Increasing pressure to lower greenhouse gas emissions, decrease the consumption of non-renewable resources and implement circular economy principles has accelerated the search for construction materials derived from renewable resources and agricultural by-products [1,2]. In this context, natural fibre-reinforced polymer biocomposites offer a practical route to combine polymer matrices with lignocellulosic reinforcement derived from renewable biomass [3].

Interest in these materials extends beyond their environmental benefits. Compared with conventional glass- or carbon-fibre-reinforced composites, natural fibre-reinforced polymer biocomposites generally exhibit lower density, reduced embodied energy and improved end-of-life management through recycling or biodegradation [2,4,5,6,7]. Fibres obtained from rapidly renewable crops, including hemp, flax, jute, kenaf, bamboo and wheat straw, have therefore attracted considerable attention for use in structural panels, insulation systems, acoustic elements and prefabricated building components [8,9,10,11]. Their favourable strength-to-weight ratio, widespread availability and relatively low production costs further support their increasing use in sustainable construction. Despite these advantages, maintaining service durability under humid service conditions remains one of the principal challenges limiting broader implementation of these materials [12,13,14].

Despite their recognised environmental advantages, natural fibre-reinforced polymer composites continue to face significant challenges related to service durability. The primary limitation arises from the lignocellulosic structure of plant fibres, which contains numerous hydroxyl (-OH) groups responsible for their strong affinity for water [15]. Moisture absorption not only alters the dimensional stability of the fibres but also creates conditions favourable for biological colonisation, particularly during prolonged exposure to damp environments. In buildings, such conditions may develop as a consequence of thermal bridges, water leakage or vapour condensation within the building envelope. Once sufficient moisture is available, lignocellulosic fibres become a suitable substrate for microorganisms, which utilise their organic constituents as nutrient sources and initiate progressive biodeterioration [16,17].

Growing interest in bio-based construction materials is also driven by their potential contribution to decarbonising the built environment. Recent studies have shown that replacing conventional construction materials with biomaterials can substantially reduce embodied carbon emissions while supporting circular economy strategies [18,19]. Nevertheless, the increasing use of natural fibre-reinforced biocomposites has outpaced our understanding of their long-term performance. Although considerable progress has been achieved in material design and manufacturing technologies, important questions remain regarding durability under in-use conditions, where cyclic variations in humidity, temperature and microbial activity act simultaneously rather than in isolation [2].

The recent literature further emphasises that the sustainability potential of natural-fibre composites extends beyond the substitution of synthetic reinforcement and includes opportunities associated with renewable feedstocks, lower material-related environmental burdens and circular material strategies. However, these benefits depend strongly on fibre processing, matrix selection, manufacturing route, durability and the adopted end-of-life scenario [20,21].

The recent literature confirms the growing role of natural fibres in sustainable construction, including their application in composite panels, insulation systems and other building components. However, broader implementation continues to be constrained by moisture sensitivity, variability of natural raw materials and uncertainties concerning long-term durability under service conditions [22].

Previous studies have extensively investigated the mechanical and physical performance of natural fibre-reinforced materials, including the effects of fibre incorporation and modification on strength, dimensional stability, moisture transport and thermal behaviour [3,23]. For example, Ref. [23] demonstrated that hydrothermally treated wood fibres incorporated into cement mortars improved dimensional stability, flexural strength, thermal insulation properties and capillary-water absorption behaviour, particularly during ageing [23]. However, considerably less attention has been devoted to the coupled effects of fungal biodeterioration, moisture transport and progressive changes in the mechanical and thermal performance of polymer-based natural-fibre composites under long-term exposure conditions. Far less attention has been devoted to the coupled interactions between moisture transport, biodeterioration, microstructural evolution and the resulting changes in both structural integrity and thermal insulation performance. The present review addresses this gap by critically evaluating the mechanisms governing biodeterioration of natural fibre-reinforced polymer biocomposites, the factors controlling their service durability, and the effectiveness of environmentally friendly strategies developed to mitigate biological degradation under service conditions and by discussing current limitations of standardised durability assessment methods for bio-based construction composites.

Recent developments increasingly position natural-fibre composites within a broader circular-economy framework, in which renewable feedstocks, reduced dependence on synthetic reinforcement, resource efficiency and end-of-life management are considered alongside material performance [20].

Despite substantial progress in fibre modification, interfacial engineering and composite processing, recent reviews continue to identify moisture sensitivity, fibre–matrix incompatibility and durability-related limitations as major barriers to the wider engineering implementation of natural-fibre composites [11,24]. These limitations are particularly important in construction applications, where materials are expected to retain their functional properties under prolonged and variable environmental exposure.

Although recent reviews provide comprehensive assessments of natural-fibre composite processing, sustainability, interfacial modification and engineering applications, the coupled relationships between fungal biodeterioration, moisture transport, microstructural deterioration and the resulting mechanical and thermal performance remain comparatively underexplored [11,20,21].

### Literature Search and Review Methodology

This review was conducted as a structured critical literature review focused on the biodeterioration, hygrothermal durability and resulting mechanical and thermal performance of natural fibre-reinforced polymer biocomposites relevant to construction applications. Scopus and Web of Science were used as the principal bibliographic databases, supplemented by searches using ScienceDirect and Google Scholar. The search primarily covered peer-reviewed journal articles published up to 2026, while earlier seminal studies were retained where they provided fundamental information on natural-fibre chemistry, moisture transport, fungal degradation mechanisms, established durability-testing methods or other concepts essential to the interpretation of the reviewed literature.

Search terms were combined using Boolean operators and included terms related to: (i) materials (“natural fibre composite”, “plant fibre composite”, “biocomposite”, “natural fibre-reinforced polymer”, “hemp composite”, “flax composite”, “jute composite”); (ii) degradation mechanisms (“biodeterioration”, “fungal degradation”, “fungal colonisation”, “mould growth”, “biodegradation”, “microbial colonisation”); (iii) environmental exposure (“moisture”, “water absorption”, “hygrothermal ageing”, “wet–dry cycling”, “natural weathering”); and (iv) functional performance (“mechanical properties”, “strength”, “stiffness”, “thermal conductivity”, “insulation”, “durability”, “service life”). Terms within each thematic group were combined using OR, whereas material-related terms were combined using AND with at least one of the three thematic groups. Additional targeted supplementary searches were conducted to broaden coverage of protection strategies, recyclability and life-cycle assessment, service-life prediction, and recent developments and emerging approaches.

Studies were included when they addressed at least one of the following aspects: natural fibre-reinforced polymer composites with potential relevance to construction; moisture- or hygrothermal-induced degradation; fungal or microbial deterioration of lignocellulosic reinforcement; changes in mechanical, microstructural or thermal properties; durability-enhancing treatments; or methods relevant to long-term service-life assessment. Publications were assessed for relevance to the review scope, methodological adequacy and availability of relevant data. Priority was given to experimental studies providing quantitative data on exposure conditions and property changes, particularly where unaged or reference values enabled calculation of relative degradation. Review articles were used primarily to establish broader research trends and identify additional primary studies. Studies dealing exclusively with unrelated biomedical applications, food packaging or composites without relevance to construction durability were excluded from the core synthesis.

**Figure 1:**
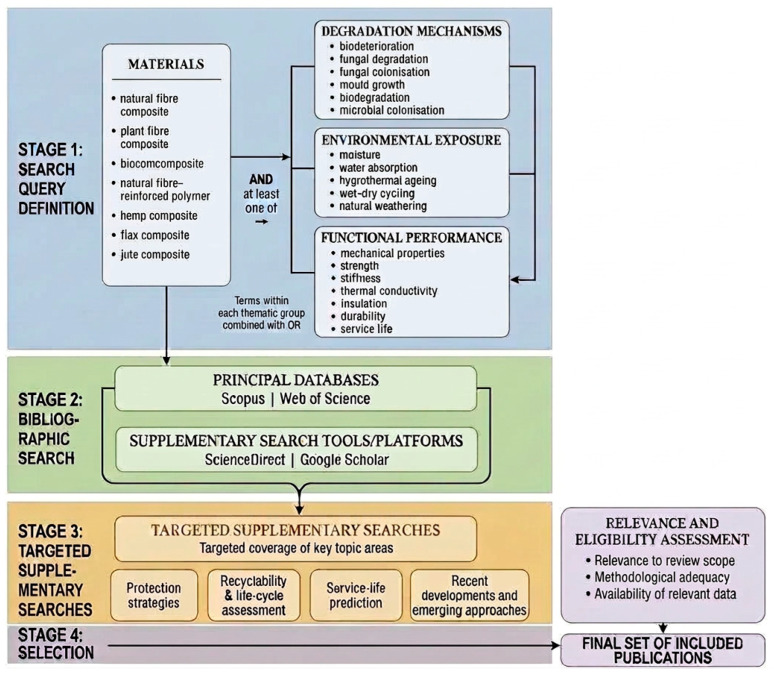
Structured literature search and publication selection workflow applied in this review. Terms within each thematic group were combined using the Boolean operator OR, whereas material-related terms were combined with at least one thematic group using AND. Targeted supplementary searches were used to broaden coverage of protection strategies, recyclability and life-cycle assessment, service-life prediction, and recent developments and emerging approaches.

The structured literature search and publication selection workflow is summarised in Figure 1. The procedure comprised search-query definition, bibliographic searching in principal databases and supplementary search platforms, targeted supplementary searches addressing specific thematic areas, and relevance and eligibility assessment prior to establishing the final set of included publications.

Because the reviewed studies employed heterogeneous material formulations, exposure protocols and test methods, a formal meta-analysis was not considered appropriate. Instead, quantitative results were normalised, where possible, relative to the corresponding unaged or reference state to facilitate cross-study comparison. Mechanical and thermal data were interpreted separately according to the dominant exposure mechanism, distinguishing moisture/hygrothermal ageing from biologically mediated deterioration. This approach was used to avoid attributing moisture-induced property changes directly to fungal activity where biological effects had not been independently quantified.

The search emphasised the literature published between 2015 and 2026, with particular attention to studies from 2022 to 2026; earlier publications were included when they represented seminal or methodologically important contributions.

## 2. Structure–Property Relationships in Natural Fibre-Reinforced Biocomposites

The performance of natural fibre-reinforced polymer biocomposites is governed not only by the intrinsic properties of their individual constituents but also, and often more importantly, by the interactions that develop at the fibre–matrix interphase. The reinforcing fibres, polymer matrix and interfacial transition zone function as an integrated system, in which the characteristics of each component influence the overall behaviour of the composite. Consequently, the chemical composition of both phases, together with the quality of interfacial adhesion, determines the efficiency of stress transfer, moisture resistance, dimensional stability and service durability under service conditions [3].

### 2.1. Characteristics of Natural Fibres

Natural fibres used as reinforcement in polymer biocomposites exhibit considerable variability in their chemical composition, which largely determines their mechanical performance, moisture behaviour and resistance to biological degradation [8,25]. Although cellulose, hemicelluloses and lignin are the principal constituents of all lignocellulosic fibres, their relative proportions differ substantially among plant species, resulting in significant differences in engineering performance and service durability [25].

Cellulose forms the main structural framework of plant fibres and is primarily responsible for their tensile strength and stiffness. In contrast, hemicelluloses possess a highly amorphous structure and exhibit pronounced hygroscopicity, making them particularly susceptible to hydrolysis and enzymatic degradation. Lignin functions as a natural binding polymer within the cell wall, increasing structural rigidity while generally improving resistance to microbial attack [7]. Owing to their lower chemical stability, hemicelluloses are typically the first constituents degraded during fungal colonisation. Their decomposition facilitates moisture penetration into the fibre interior and accelerates the subsequent deterioration of the remaining lignocellulosic structure [26].

In addition to chemical composition, the durability of natural fibre-reinforced biocomposites is strongly influenced by the geometrical characteristics and spatial arrangement of the reinforcement. Fibre aspect ratio affects stress-transfer efficiency and fibre–matrix interfacial characteristics. Higher aspect ratios can improve mechanical reinforcement when sufficient fibre wetting and impregnation are achieved, whereas inadequate impregnation may promote interfacial defects and moisture-sensitive pathways [27,28]. Fibre volume fraction has a similarly non-linear effect. Increasing fibre content generally enhances reinforcement efficiency up to an optimum level, whereas excessive fibre loading may hinder complete matrix wetting, promote fibre agglomeration and increase void content, potentially facilitating moisture retention [29]. Fibre orientation further controls mechanical anisotropy and the spatial distribution of fibre–matrix interfaces. Aligned architectures generally provide efficient directional load transfer, whereas random or multilayered arrangements modify stress distribution and the distribution of potential interfacial defects [29]. These architectural differences may also influence moisture-transport pathways, although this relationship remains less systematically quantified in natural fibre/polymer composites. Fibre architecture can additionally influence thermal performance. A recent numerical and experimental study demonstrated that fibre aspect ratio and orientation modify heat-transfer pathways and effective thermal conductivity, indicating that reinforcement geometry can affect not only mechanical anisotropy but also the insulation performance of natural fibre/polymer composites [30]. Consequently, aspect ratio, fibre volume fraction and orientation may influence biodeterioration indirectly by modifying interfacial characteristics, void structure and moisture transport, thereby affecting the accessibility of lignocellulosic reinforcement to microorganisms. These parameters should therefore be considered together with fibre chemistry when comparing the biological durability of different composite architectures. The principal characteristics of natural fibres commonly used in construction biocomposites are summarised in Table 1, including their chemical composition, density, moisture absorption capacity and relative susceptibility to biodeterioration [5,8,9,31].

The compositional values represent literature-reported ranges and may vary with plant variety, cultivation conditions, harvesting, fibre extraction and processing. The chemical composition alone should not be interpreted as a direct measure of biodeterioration susceptibility, which additionally depends on moisture availability, fibre treatment, composite architecture, matrix characteristics and exposure conditions.

The moisture affinity of natural fibres cannot be represented reliably by a single qualitative ranking because reported values depend strongly on relative humidity, temperature, fibre processing and the parameter used to quantify moisture uptake. For example, literature compilations report equilibrium moisture contents of approximately 7% for flax, 9% for hemp and 12% for jute under reference conditions, whereas experimental sorption data for kenaf and flax at 15 °C increased from approximately 12.4% and 9.4%, respectively, at 43% RH to 27.2% and 23.0% at 96% RH [32]. These results demonstrate that moisture availability and environmental exposure conditions should be considered together with fibre chemistry when assessing susceptibility to biodeterioration.

The susceptibility categories presented in Table 1 should be interpreted as a comparative engineering classification rather than as absolute or standardised measures of biological resistance. The classification was derived from the combined consideration of fibre chemical composition, moisture affinity and published observations of microbial degradation [5,8,9,31]. Hemicelluloses are comparatively hygroscopic and readily accessible to hydrolytic and microbial degradation, whereas lignin generally provides greater resistance to enzymatic attack. Consequently, fibres with similar cellulose contents may exhibit different biodeterioration behaviours because of differences in hemicellulose-to-lignin ratio, moisture sorption and anatomical structure. However, chemical composition alone cannot predict biological durability, which is additionally controlled by fibre morphology, surface treatment, composite porosity, fibre–matrix interfacial quality and exposure conditions.

Variations reported among published studies largely reflect differences in fibre origin, polymer matrices, environmental exposure conditions and experimental procedures used to assess biodeterioration. Consequently, direct comparison of results should be undertaken with caution, particularly when studies employ different testing methodologies or exposure scenarios.

### 2.2. Polymer Matrices and the Interphase Zone

The long-term performance of construction biocomposites depends not only on the properties of the reinforcing fibres but also on the characteristics of the polymer matrix and, most importantly, on the quality of the fibre–matrix interphase. Construction biocomposites are currently manufactured using a broad range of polymer matrices, including conventional thermosetting resins such as epoxy, polyester and polyurethane, as well as recycled hydrophobic thermoplastics, including recycled polypropylene (r-PP), polyethylene (r-PE) and poly(vinyl chloride) (r-PVC). Increasing attention has also been directed towards biodegradable polymers, particularly polylactic acid (PLA) and polyhydroxyalkanoates (PHA) because of their lower environmental impact and their potential contribution to more sustainable material systems [1].

Despite this diversity of polymer matrices, achieving a durable fibre–matrix interphase remains one of the principal challenges in biocomposite engineering. Natural lignocellulosic fibres are inherently hydrophilic, whereas most polymer matrices exhibit hydrophobic behaviour. This mismatch in surface chemistry limits interfacial compatibility and often results in incomplete fibre wetting during processing. As a consequence, microvoids, interfacial gaps and local delamination defects may develop within the composite structure, reducing both mechanical integrity and service durability [33].

The extent and distribution of these structural discontinuities are largely governed by the effectiveness of fibre impregnation and the performance of coupling agents used during manufacturing. Once formed, interfacial defects act as preferential pathways for moisture transport, allowing water to penetrate deep into the composite. The resulting increase in local moisture content not only accelerates fibre swelling and interfacial degradation but also creates conditions that support microbial colonisation, thereby promoting progressive biodeterioration [17,33,34].

Considerable research has therefore focused on improving fibre–matrix compatibility through chemical and physical surface modification techniques. Among the most widely adopted industrial solutions for non-polar polymer matrices is maleic anhydride-grafted polypropylene (MAPP). In this system, maleic anhydride groups react with hydroxyl groups present on the cellulose surface to form ester bonds, while the polypropylene backbone becomes physically entangled with the surrounding matrix. This dual interaction enhances interfacial adhesion, improves stress transfer and reduces the formation of defects that facilitate moisture ingress into the composite.

Silane coupling agents represent an alternative approach to improving fibre–matrix compatibility. Following hydrolysis in aqueous–alcoholic media, silane molecules form reactive silanol groups that condense on the fibre surface, producing stable Si–O–Si and Si–O–C covalent bonds. This surface modification effectively blocks a substantial proportion of the accessible hydroxyl groups, thereby reducing interfacial moisture transport and limiting the conditions favourable for microbial colonisation.

The effectiveness of coupling agents, however, depends on several material- and process-related factors. In the case of MAPP, the degree of maleic anhydride grafting, polymer molecular weight and fibre content all influence the quality of interfacial bonding. Composites containing high proportions of lignocellulosic reinforcement often benefit from combining different modification strategies, such as alkaline fibre treatment followed by the application of coupling agents, resulting in improved fibre wetting and more stable interfacial adhesion.

Representative applications of natural fibre-reinforced polymer biocomposites in the construction sector are summarised in Table 2, which presents commonly used reinforcement fibres, polymer matrices or binders, their principal functional properties and representative literature sources.

The examples presented in Table 2 demonstrate the versatility of natural fibre-reinforced biocomposites across a wide range of construction applications, including structural panels, fibre-reinforced composite elements and thermal insulation systems. Although the selection of the polymer matrix is dictated primarily by functional and processing requirements, long-term performance ultimately depends on the stability of the fibre–matrix interphase. Durable interfacial bonding ensures efficient stress transfer while simultaneously restricting moisture ingress into the composite.

Recent reviews further confirm that the mechanical performance of natural fibre-reinforced polymer composites is strongly influenced by fibre characteristics, matrix selection and fibre–matrix interfacial quality, while moisture absorption remains a major factor affecting mechanical performance and durability [38]. Under cyclic environmental exposure, repeated fibre swelling and shrinkage progressively weaken the interface, leading to the formation of additional microcracks and a gradual loss of mechanical integrity.

These degradation processes are further intensified by differences in hygroscopic behaviour and coefficients of thermal expansion between natural fibres and polymer matrices. Repeated fluctuations in temperature and relative humidity generate cyclic stresses at the interface, promoting progressive debonding and delamination throughout the service life of the composite. As a result, the material becomes increasingly permeable to moisture and progressively more susceptible to biologically induced deterioration.

### 2.3. Classification of Construction Applications and Biodeterioration Risk

From a durability perspective, natural fibre-based biocomposites should not be considered as a single homogeneous group of construction materials. Their susceptibility to biodeterioration and the consequences of biological damage depend strongly on the function of the building component, the magnitude of mechanical loading, exposure to moisture and the accessibility of the material to inspection or replacement. For the purpose of durability assessment, construction applications can therefore be broadly classified into three functional categories: load-bearing, semi-load-bearing and non-load-bearing/insulating components. This classification is particularly relevant because a similar degree of fungal colonisation may have markedly different engineering consequences depending on the function performed by the affected component. Recent reviews confirm the expanding range of natural-fibre applications in building components, from composite elements to thermal and acoustic insulation systems [22,39]. The application-oriented classification and the corresponding biodeterioration risks are summarised in Table 3.

Load-bearing applications represent the most durability-sensitive category because biological deterioration may directly affect structural reliability. Potential applications include structural sandwich panels, fibre-reinforced profiles, decking systems and other composite elements designed to transfer sustained or cyclic loads. In these components, moisture penetration and fungal degradation may progressively weaken natural fibres and the fibre–matrix interface, resulting in reductions in stiffness, strength and fatigue resistance. Interfacial microcracking may additionally accelerate moisture ingress and create localised zones favourable to microbial colonisation. Consequently, even relatively limited biodeterioration may be critical when it occurs in highly stressed regions or in components with limited possibilities for inspection [6,15,17,34].

Semi-load-bearing applications include façade and cladding panels, partition systems, ceiling elements, door components and prefabricated enclosure panels, where biocomposites contribute to stiffness, dimensional stability and local load transfer without functioning as primary structural members. In this category, biodeterioration may not immediately compromise global structural safety but can progressively reduce serviceability through swelling, warping, cracking, delamination and loss of surface integrity. Façade and envelope applications are particularly sensitive because cyclic wetting, condensation and seasonal variations in temperature and relative humidity may repeatedly activate moisture transport and create conditions favourable to fungal growth. The durability assessment of such components should therefore combine mechanical criteria with dimensional stability, moisture resistance and surface biodeterioration indicators [13,33,35,40].

Non-load-bearing and thermal-insulation applications constitute a distinct risk category because functional failure is governed primarily by changes in moisture behaviour, thermal conductivity, dimensional stability and indoor biological contamination rather than by loss of load-bearing capacity. Typical applications include insulation boards, lightweight infill panels and other porous bio-based components incorporated into wall, roof and floor assemblies. Their high porosity and hygroscopic lignocellulosic content promote moisture buffering but may also prolong local moisture retention under unfavourable hygrothermal conditions. Fungal colonisation may therefore contribute to changes in pore structure and moisture transport, while increased water content directly increases thermal conductivity and reduces insulation efficiency [36,37,41,42,43,44]. In components facing the indoor environment, microbial growth may additionally affect indoor air quality through the release of spores, fragments and microbial volatile organic compounds (MVOCs), making biological durability relevant not only to material performance but also to occupant exposure [45,46].

The comparison demonstrates that biodeterioration risk should be evaluated according to the functional role of the component rather than solely on the basis of material susceptibility to microbial growth. For load-bearing components, the critical consequence is the progressive loss of mechanical reliability; for semi-load-bearing components, dimensional stability, surface integrity and durability under cyclic environmental exposure become dominant; and for insulating components, moisture retention, thermal-performance deterioration and potential indoor microbial exposure are of primary concern. This application-oriented framework therefore provides a basis for selecting appropriate durability indicators and testing procedures for different classes of construction biocomposites.

## 3. Biodeterioration Mechanisms

Moisture availability is the primary prerequisite for the initiation of biodeterioration in natural fibre-reinforced biocomposites. Microbial colonisation begins only when the moisture content and water activity within the material exceed the threshold required for fungal spore germination and subsequent microbial growth [47,48]. Although this threshold depends on several factors, including substrate characteristics, fungal species, exposure duration, temperature and local environmental conditions, prolonged exposure to elevated humidity is consistently recognised as the dominant factor triggering biodeterioration in building materials [45,46,49]. As moisture progressively accumulates within the pore network, the material gradually loses its ability to dry efficiently and maintain hygrothermal equilibrium. Persistent wet conditions subsequently create an environment that favours microbial proliferation and accelerates the onset of biological degradation [47,49,50].

Biodeterioration should not be regarded as a single degradation event but rather as a progressive sequence of interconnected processes. Moisture uptake initiates microbial colonisation of the material surface, followed by biofilm formation and the production of extracellular enzymes capable of degrading lignocellulosic constituents. Biofilm formation begins with the adhesion and germination of fungal spores on a sufficiently moist material surface, followed by the development of hyphae and an extracellular matrix that facilitates attachment and moisture retention. Within this local microenvironment, fungi secrete extracellular hydrolytic and oxidative enzymes, including cellulases, hemicellulases and, depending on the fungal species, lignin-modifying enzymes. These enzymes depolymerise lignocellulosic constituents into smaller molecules that can be assimilated by fungal cells, progressively weakening the fibre structure and increasing its susceptibility to further moisture penetration and mechanical damage [48,51,52]. As degradation advances, these processes progressively weaken the fibre–matrix interphase, alter the internal microstructure and ultimately impair both the structural integrity and functional performance of the composite.

### 3.1. Structural Degradation

**Figure 2:**
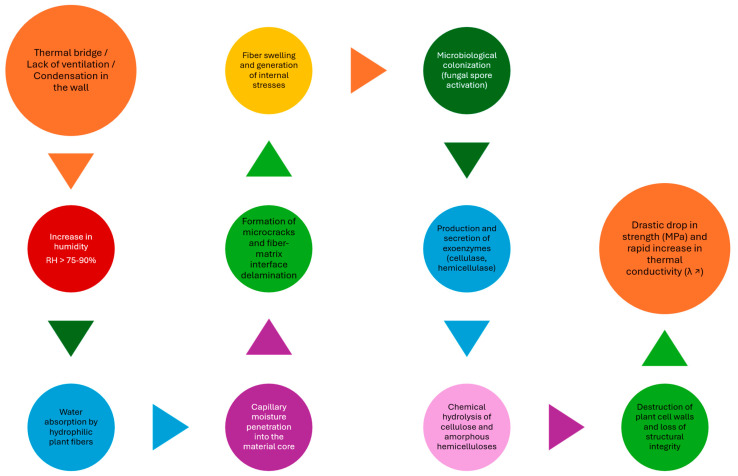
Cascade of physicobiological biodeterioration of a construction biocomposite. Prepared by the author.

Structural degradation of natural fibre-reinforced biocomposites results from a sequence of coupled physicochemical and biological processes rather than from a single deterioration mechanism. As moisture accumulates within the material, progressive changes develop at the fibre–matrix interphase, initiating a cascade of degradation events that ultimately compromise the integrity and functionality of the composite. The principal stages of this process are illustrated schematically in Figure 2.

As shown in Figure 2, the earliest changes are associated with moisture-induced swelling of lignocellulosic fibres and repeated hygrothermal stresses acting at the fibre–matrix interphase. These processes gradually generate microstructural discontinuities, including interfacial debonding, microvoids and microcracks, which facilitate moisture transport into the interior of the composite [40,53]. Once sufficient moisture has accumulated, the material becomes increasingly susceptible to microbial colonisation, initiating biochemical degradation and accelerating the cumulative damage of its internal structure [54].

A key stage of biodeterioration involves the enzymatic decomposition of cellulose by filamentous fungi. These microorganisms produce a coordinated cellulolytic enzyme system that acts synergistically on lignocellulosic substrates. Endoglucanases (EC 3.2.1.4) initiate degradation by randomly cleaving internal β-1,4-glycosidic bonds within the amorphous regions of cellulose, thereby reducing polymer chain length. Exoglucanases (cellobiohydrolases, EC 3.2.1.91) subsequently release cellobiose units from cellulose chain ends, whereas β-glucosidases (EC 3.2.1.21) hydrolyse cellobiose to glucose, completing cellulose depolymerisation [48].

Enzymatic degradation is accompanied by oxidative reactions that further accelerate structural deterioration. Brown-rot fungi produce low-molecular-weight metabolites capable of initiating the Fenton reaction, resulting in the generation of highly reactive hydroxyl radicals [51]:

Fe^2+^ + H_2_O_2_ → Fe^3+^ + OH^−^ + ∙OH, (1)

Hydroxyl radicals readily diffuse through the pore network, attacking cellulose and hemicellulose by cleaving glycosidic bonds independently of enzymatic activity. This oxidative pathway substantially weakens the lignocellulosic framework, increases fibre porosity and facilitates deeper penetration of fungal enzymes, thereby accelerating degradation of the fibre–matrix interphase.

The relative importance of individual degradation mechanisms depends largely on the fungal species colonising the material. Brown-rot fungi preferentially degrade polysaccharides through oxidative processes associated with the Fenton reaction, whereas white-rot fungi possess a broader enzymatic system, including laccases, lignin peroxidases and manganese peroxidases, enabling the simultaneous degradation of lignin, cellulose and hemicellulose [51,52]. As a consequence, white-rot fungi generally induce more extensive disruption of the lignocellulosic cell wall, leading to more severe deterioration of the composite microstructure.

Collectively, these physicochemical and biological processes progressively reduce the effectiveness of stress transfer across the fibre–matrix interphase, increase moisture permeability and accelerate the loss of mechanical integrity [38,55]. Structural degradation therefore represents a critical stage linking microbial activity with the long-term deterioration of engineering performance in natural fibre-reinforced polymer biocomposites.

### 3.2. Biological Factors

The initiation and progression of biodeterioration depend not only on environmental conditions but also on the biological characteristics of the microorganisms colonising the material. Among them, filamentous fungi are regarded as the principal agents responsible for the deterioration of natural fibre-reinforced biocomposites because of their ability to utilise lignocellulosic constituents as nutrient sources [17,56]. The extent of fungal colonisation is governed by the combined influence of moisture availability, pore structure, oxygen accessibility and the duration of favourable environmental conditions. Consequently, materials exhibiting high moisture retention and open pore networks generally provide more suitable habitats for microbial growth [45,46].

The fungal species most frequently associated with the deterioration of natural fibre-reinforced biocomposites are summarised in Table 4. Although many of these microorganisms develop under comparable environmental conditions, they differ considerably in their moisture requirements, growth dynamics and colonisation strategies [46]. These biological differences largely determine both the rate of microbial development and the severity of deterioration observed in construction materials.

The comparison presented in Table 4 illustrates that fungal species affect biocomposites through different modes of colonisation. Some microorganisms, such as *Cladosporium cladosporioides*, predominantly develop on the material surface, leading mainly to biofilm formation and aesthetic deterioration. Others, including *Trichoderma viride* and *Chaetomium globosum*, readily penetrate the lignocellulosic structure and promote extensive degradation of the reinforcing fibres. Species belonging to the genera *Aspergillus* and *Penicillium* are particularly important because of their rapid growth under elevated humidity and their frequent occurrence in building environments, making them among the most common initiators of biodeterioration in construction biocomposites [17,57,58,59].

The biological activity of filamentous fungi is closely linked to changes in the internal environment of the composite. As microbial colonies expand, the developing mycelial network facilitates further moisture retention and creates local microenvironments that favour continued microbial growth. This positive feedback accelerates biodeterioration, particularly in regions where moisture persists for prolonged periods [45,46]. Consequently, fungal colonisation should be regarded as a dynamic process that continuously modifies the internal structure of the composite rather than merely as surface contamination.

Differences in fungal physiology also explain the considerable variability reported in the literature regarding the durability of natural fibre-reinforced biocomposites. Growth rate, moisture tolerance and substrate utilisation differ substantially among species, making direct comparison of experimental studies difficult unless environmental conditions and microbial inocula are carefully standardised. These observations emphasise the importance of considering both material characteristics and biological factors when assessing the service durability of bio-based construction composites. This variability further emphasises the need for standardised biological exposure protocols; although ISO 846:2019 provides established procedures for evaluating the effects of microorganisms on plastics, its scope is not specifically designed to quantify biodeterioration-induced functional deterioration in natural fibre-reinforced construction composites [60].

## 4. Degradation of Mechanical and Thermal Properties

The deterioration of mechanical and thermal performance represents one of the most significant consequences of biodeterioration in natural fibre-reinforced polymer biocomposites. As biological degradation progresses, changes occurring within the lignocellulosic fibres and at the fibre–matrix interphase gradually compromise the structural continuity of the composite. The combined effects of fibre degradation, interfacial debonding and microstructural damage reduce the efficiency of stress transfer while simultaneously modifying heat and moisture transport through the material [15,17,61].

The extent of property degradation is governed by the interaction of multiple factors rather than by biological activity alone. Fibre type, polymer matrix characteristics, interfacial adhesion, moisture uptake, exposure conditions and the duration of microbial colonisation all contribute to the rate and severity of deterioration. Their combined influence largely explains the variability reported in the literature regarding changes in the mechanical behaviour and thermal performance of construction biocomposites [15,61].

### 4.1. Deterioration of Mechanical Properties

Moisture is widely recognised as one of the primary factors governing the deterioration of mechanical performance in natural fibre-reinforced biocomposites. Water absorbed by lignocellulosic fibres induces fibre swelling, weakens the fibre–matrix interphase and promotes the initiation of microstructural defects. As these changes accumulate, crack propagation accelerates, progressively reducing the load-bearing capacity of the composite [11,40].

Published studies consistently demonstrate that deterioration of mechanical properties follows a common degradation pathway initiated by moisture uptake. Fibre swelling, progressive debonding at the fibre–matrix interphase and the formation of microcracks are repeatedly identified as the principal mechanisms responsible for reductions in stiffness and strength, although the severity of deterioration depends on fibre type, matrix composition and environmental exposure conditions [15,17,40,61].

Investigations on hemp fibre-reinforced polyester composites demonstrated that increasing fibre content resulted in greater moisture absorption, which was accompanied by progressive reductions in both tensile and flexural strength [15]. These changes were primarily attributed to deterioration of the fibre–matrix interphase and microcrack formation induced by fibre swelling. The degradation became considerably more pronounced after exposure at 100 °C than under ambient laboratory conditions, demonstrating the combined influence of moisture and temperature on composite durability [15].

Similar observations have been reported for flax fibre-reinforced composites. Detailed microstructural analyses revealed that fibre orientation, local agglomeration and non-uniform reinforcement distribution generate stress concentrations that subsequently act as preferential sites for crack initiation [62]. Although these defects often originate during manufacturing, they may remain dormant until activated by prolonged moisture exposure and biological attack [17,62].

The contribution of biological degradation has been clearly demonstrated in studies involving the cellulolytic fungus *Chaetomium globosum* [17]. After twenty-eight days of exposure under high-humidity conditions, flax and hemp mats, together with flax/PP and hemp/PP composites, exhibited measurable dry-mass losses, a reduction in elastic modulus and extensive fungal colonisation. Scanning electron microscopy further confirmed cumulative damage of fibre surfaces and disruption of the composite microstructure [17].

Differences among fungal species are equally important: recent WPC studies demonstrate that soft-rot and wood-decaying fungi may produce substantially different levels of mass loss and material deterioration, confirming that fungal species must be treated as an independent experimental variable in comparative durability assessments [17,57,58,59].

Recent review papers indicate that prolonged moisture exposure may reduce tensile strength by as much as 40%, while elastic modulus commonly decreases by 20–30%, depending on fibre type, composite composition and environmental conditions [40]. Similar conclusions were reported in [6], which identified moisture ingress as the principal factor initiating degradation in natural-fibre composites. Water absorption promotes fibre swelling, increases porosity and progressively weakens fibre–matrix adhesion, leading to a gradual decline in mechanical performance [34]. Consequently, prolonged service under humid environmental conditions may substantially reduce the operational lifetime of construction biocomposites.

Table 5 summarises representative studies describing changes in the mechanical and physical properties of selected natural fibre-reinforced biocomposites following moisture exposure or biodeterioration.

The studies summarised in Table 5 reveal a consistent deterioration pattern despite differences in materials and experimental methodologies. The greatest reductions in mechanical performance are generally observed in flax- and hemp-reinforced composites exposed to prolonged humid conditions. In most cases, degradation is associated with weakening of the fibre–matrix interphase accompanied by cumulative damage of lignocellulosic constituents.

Although all investigations report a decline in mechanical performance, the magnitude of deterioration differs considerably because several degradation mechanisms may operate simultaneously. Hydrothermal ageing is dominated by moisture sorption, fibre swelling and gradual loss of interfacial adhesion [15,40,61]. Under biological exposure, these physical processes are accompanied by enzymatic degradation of cellulose and hemicellulose, producing irreversible structural damage within the reinforcing fibres and resulting in more severe reductions in mechanical properties [17,48,51].

The degradation behaviour is also influenced by the characteristics of the polymer matrix. Thermosetting composites based on unsaturated polyester exhibit deterioration mechanisms that differ from those observed in polypropylene- or polylactic acid-based systems reinforced with natural fibres [15,61,62]. Matrix chemistry affects moisture transport, susceptibility to hydrolysis and resistance to environmental ageing, making direct quantitative comparison between different composite systems difficult [6,40,61].

Additional variability reported in the literature reflects differences in ageing protocols, exposure duration, relative humidity, temperature and mechanical testing procedures. Consequently, percentage reductions in strength or stiffness reported by different authors should always be interpreted within the context of the specific experimental methodology employed [6,40,61].

To facilitate comparison between published studies, Table 5 presents representative data illustrating the influence of moisture exposure and biodeterioration on the mechanical and thermal behaviour of natural fibre-reinforced biocomposites under different environmental conditions.

Recent experimental studies provide a more detailed quantitative picture of moisture-induced mechanical deterioration. Ref. [63] investigated non-woven flax/PLA composites subjected to controlled hygroscopic ageing and water immersion. Exposure at 98% RH reduced tangent modulus by 23.0% and ultimate strength by 26.7%, whereas water immersion produced larger reductions of 33.8% and 37.4%, respectively. The comparison demonstrates a clear exposure-severity effect: direct water contact caused substantially greater deterioration than high-humidity conditioning, reflecting increased fibre swelling, porosity development and matrix microcracking.

A different degradation pattern was reported in [64] for woven flax/PLA composites exposed to 40 °C and 100% RH for up to 42 days. Average stiffness decreased by approximately 52–54% within the first 24 h and by 69–72% after 4 days, followed by relative stabilisation, whereas tensile strength decreased more gradually, from approximately 70 MPa to 50 MPa after 42 days (~29%). These results indicate that stiffness may respond considerably earlier than ultimate strength to hygrothermal degradation and may therefore represent a more sensitive early indicator of fibre–matrix interfacial deterioration.

Taken together, these results demonstrate that mechanical degradation cannot be represented adequately by a single strength-loss parameter. The relative sensitivity of individual mechanical properties varies substantially with fibre type, matrix chemistry, composite architecture and exposure mechanism. In flax/PLA composites, stiffness may deteriorate considerably earlier and more strongly than ultimate strength, indicating early loss of fibre–matrix load-transfer efficiency [64]. Under combined UV–thermal–moisture ageing, impact strength may be affected more severely than tensile or flexural properties [65]. These contrasting responses demonstrate that durability assessment should include multiple mechanical indicators rather than relying exclusively on ultimate tensile strength.

More recent studies confirm that the magnitude of moisture-induced deterioration varies substantially among natural fibre–polymer systems and cannot be extrapolated directly from one composite architecture to another. Accelerated water-immersion studies further demonstrate that moisture durability is strongly influenced by material composition, composite architecture and the presence of interfacial regions and voids that facilitate water ingress. In large-format additively manufactured composites, wood-flour-reinforced PLA exhibited substantially greater moisture uptake and more progressive degradation than the investigated synthetic-fibre-reinforced polymer systems, while mechanical deterioration was particularly pronounced in directions containing a greater number of interlayer interfaces and voids [66]. More broadly, differences in moisture-induced mechanical deterioration among natural-fibre composites have been attributed to fibre type, matrix characteristics, fibre content, surface treatment and the resulting fibre–matrix interfacial stability [40]. Consequently, comparable moisture exposure does not necessarily result in comparable mechanical deterioration because fibre swelling, matrix plasticisation, interfacial debonding and irreversible microcracking may contribute to the observed loss of performance to different extents [40].

The influence of matrix chemistry is particularly evident in natural fibre-reinforced hybrid polymer composites. Authors in [67] investigated flax–glass hybrid FRP composites with epoxy (EP) and polyurethane (PUR) matrices subjected to hygrothermal and natural weathering exposure. Depending on the exposure conditions, the tensile-strength retention of EP-based composites ranged from 65.4 to 88.0%, corresponding to losses of approximately 12.0–34.6%, whereas PUR-based composites retained 80.7–94.1% of their initial strength, corresponding to substantially lower losses of approximately 5.9–19.3%. Despite these changes in strength, tensile modulus was comparatively insensitive to the applied exposure conditions. The results demonstrate that the polymer matrix plays a decisive role in controlling moisture-induced degradation: higher moisture uptake in the epoxy-based system resulted in greater loss of tensile performance than in the polyurethane-based composite. This comparison also shows that the relative sensitivity of strength and stiffness is not universal but depends on composite architecture and matrix chemistry [67].

The importance of the exposure mechanism is further demonstrated by recent combined-ageing experiments. Ref. [65] subjected hemp fibre-reinforced polymer composites to coupled UV–thermal–moisture ageing and reported reductions in tensile strength of 9.57–22.12% and considerably larger losses in impact strength of 38.68–46.03%, while flexural strength remained comparatively stable. Microscopic observations revealed surface whitening, progressive microcracking and fibre exposure. These results demonstrate that different mechanical properties may exhibit markedly different sensitivities to the same ageing environment. Consequently, assessment based exclusively on tensile strength may underestimate the overall deterioration of a biocomposite, particularly when damage mechanisms affect impact resistance or the fibre–matrix interface before substantial loss of flexural load-bearing capacity occurs [65].

However, moisture-induced ageing should not be considered equivalent to biodeterioration. Hygrothermal exposure primarily initiates physical and physicochemical mechanisms, including fibre swelling, matrix plasticisation, interfacial debonding and microcracking, whereas fungal colonisation introduces an additional biological degradation pathway involving enzymatic decomposition of lignocellulosic constituents. Ref. [17] demonstrated that fungal exposure of hemp- and flax-based composites affected both fibre integrity and mechanical performance, confirming that biological activity may contribute to irreversible structural deterioration beyond the effects associated with moisture alone [17]. Therefore, quantitative comparisons of durability data should distinguish between moisture-driven degradation and biologically mediated deterioration rather than treating them as a single ageing mechanism [17]. Direct quantitative evidence for fungal-induced mechanical deterioration of natural fibre–polymer composites remains considerably more limited than data available for moisture and hygrothermal ageing. Authors in [17] compared hemp- and flax-based materials exposed to water alone, with specimens subjected to fungal deterioration, demonstrating that the presence of cellulolytic fungi produced additional changes in mass and mechanical performance that could not be attributed solely to moisture exposure [17].

Earlier coupled-exposure studies also showed that fibre size modifies both cyclic moisture uptake and fungal susceptibility, confirming that reinforcement geometry influences not only water transport but also the accessibility of lignocellulosic material to microorganisms [68]. Despite the relevance of this coupled design, comparable studies simultaneously considering reinforcement geometry, cyclic moisture exposure and controlled fungal attack remain uncommon in more recent literature.

More recent investigations on wood–polymer composites further demonstrate that biological durability is strongly influenced by prior environmental exposure. Authors in [69] showed that pre-weathering can modify the biological durability of wood–polymer composites, demonstrating that moisture behaviour and prior ageing history are important factors in subsequent resistance to fungal attack. This observation is particularly relevant for service-life assessment because biological resistance determined for unaged specimens may not fully represent the performance of composites after prolonged environmental exposure [69].

The protective role of the polymer matrix and the opposing effect of increasing lignocellulosic content have also been demonstrated in PLA-based hybrid composites. Can et al. reported that neat PLA exhibited comparatively high resistance to fungal decay, whereas increasing the proportion of wood fibre in basalt/wood-fibre-reinforced PLA composites increased susceptibility to fungal attack. The results indicate that biological durability is governed not only by matrix chemistry but also by the accessibility and volume fraction of biodegradable reinforcement. Increasing natural-fibre content therefore introduces a trade-off between renewable material content and resistance to biological deterioration, particularly when incomplete encapsulation or interfacial defects provide pathways for fungal penetration [70].

The response was strongly dependent on material architecture: loose natural-fibre mats were more susceptible to fungal attack than polymer-embedded fibres, indicating that the matrix can partially restrict microbial access to the lignocellulosic reinforcement [17]. Nevertheless, once moisture penetrates through pores, cracks or a degraded fibre–matrix interface, fungal activity may contribute to irreversible degradation of the fibre cell wall and further reduce load-transfer efficiency. This distinction is critical when interpreting published durability data, because a reduction in mechanical performance measured after fungal exposure may result from the superposition of moisture-induced physical damage and biologically mediated decomposition rather than from either mechanism acting independently [17,71].

A further limitation emerging from this comparison is the marked imbalance between the available datasets. Recent studies provide increasingly detailed quantitative information on moisture and hygrothermal ageing of natural-fibre composites, including time-dependent changes in strength, stiffness and impact resistance [40,63,64,65], whereas considerably fewer studies quantify the additional contribution of active fungal colonisation under otherwise comparable exposure conditions [56,69]. In particular, the literature reviewed here revealed only a limited number of datasets simultaneously reporting fungal development, moisture-related parameters, microstructural evolution and changes in mechanical performance. Studies additionally combining these parameters with thermal-property measurements were even scarcer. Consequently, the independent contribution of biological activity is difficult to separate quantitatively from the physical effects of moisture uptake. This represents an important methodological gap: future experimental designs should include parallel unexposed controls, moisture-only controls and biologically exposed specimens under identical hygrothermal conditions to distinguish reversible moisture-related effects from irreversible biologically induced deterioration.

Overall, the available quantitative and experimental evidence demonstrates that mechanical deterioration of natural fibre–polymer composites results from multiple interacting variables rather than from a single ageing mechanism [40]. Recent studies report tensile-strength losses ranging from less than 10% to approximately 40% under moisture, hygrothermal or combined environmental exposure, while individual properties such as stiffness or impact strength may decrease by more than 40–70% in particularly sensitive systems [40,63,64,65]. Biological durability introduces an additional level of complexity because fungal susceptibility is influenced by material composition and architecture, moisture conditions, prior ageing history and the biological agent involved [56,69]. The comparison therefore shows that degradation values reported in different studies are only meaningful when interpreted together with the corresponding material architecture and exposure protocol.

The quantitative findings reported across representative experimental studies are summarised in Table 6. The comparison highlights substantial differences in degradation magnitude among fibre–matrix systems and exposure conditions and demonstrates that moisture-induced, hygrothermal and biologically mediated deterioration should be interpreted separately because they involve partly different degradation mechanisms.

The comparison presented in Table 6 reveals that the magnitude of mechanical deterioration varies considerably among the investigated systems. Under moisture and hygrothermal exposure, reported tensile-strength losses range from less than 10% to approximately 40%, whereas substantially greater reductions may occur for other mechanical indicators. For example, stiffness loss reached 72% in hygrothermally aged flax/PLA composites, while impact-strength reductions exceeding 38% were reported under combined environmental ageing. These differences confirm that no single mechanical parameter can adequately describe durability loss. Moreover, the substantially smaller number of directly comparable quantitative datasets for fungal exposure demonstrates that the independent contribution of biological activity to mechanical deterioration remains less well quantified than moisture-induced degradation. Consequently, the numerical degradation ranges reported across the literature should be interpreted as material- and exposure-specific responses rather than as universal biodeterioration thresholds.

Recent durability research on natural-fibre-based materials and composites has increasingly extended beyond static immersion or constant-humidity conditioning towards cyclic and multi-factor environmental exposure protocols. For example, Ref. [72] applied long-term wet–dry cycling combined with UV irradiation to investigate mechanical and chemical degradation mechanisms in flax textile-reinforced recycled aggregate concrete and its constituent materials [72]. In polymer composites, Ref. [73] separately evaluated the effects of water exposure, UV weathering and aviation-related fluids on flax fibre-reinforced epoxy composites, demonstrating the particular importance of water uptake and UV-induced degradation [73]. These studies illustrate a broader methodological trend towards exposure protocols that account for multiple environmental stressors encountered during service. Nevertheless, truly coupled studies combining cyclic hygrothermal exposure with controlled fungal colonisation remain scarce for natural fibre-reinforced polymer construction composites. Most investigations still examine environmental ageing and biological deterioration separately, limiting the direct assessment of synergistic interactions between cyclic moisture loading and active microbial degradation.

### 4.2. Thermal Performance Degradation

Thermal insulation performance is one of the principal factors determining the suitability of natural fibre-reinforced biocomposites for building applications. Owing to their highly porous microstructure and the large volume of air retained within the pore system, composites reinforced with hemp, flax or straw fibres generally exhibit thermal conductivity values comparable with those of conventional insulation materials, typically ranging from 0.04 to 0.08 W/(m·K) [13,37,41]. Maintaining these favourable thermal properties throughout the service life of the material, however, remains a major challenge because heat transfer is strongly influenced by changes in moisture content and microstructure.

Moisture plays a central role in the deterioration of thermal performance. Natural lignocellulosic fibres contain numerous hydroxyl groups associated with cellulose and hemicellulose, making them inherently hygroscopic. Under conditions of elevated relative humidity or direct water exposure, moisture is readily absorbed by the fibres and progressively redistributed throughout the pore network of the composite. As air occupying the pores is gradually replaced by water, the effective thermal conductivity increases because the thermal conductivity of water is approximately twenty-five times greater than that of air. Consequently, even moderate retained moisture may lead to a measurable reduction in the insulation efficiency of bio-based construction materials [37,42].

Unlike conventional synthetic insulation products, natural fibre-reinforced biocomposites are additionally subjected to biological degradation. Once fungal colonisation has been established, enzymatic degradation of lignocellulosic constituents progressively damages the fibre cell walls, while simultaneous deterioration of the fibre–matrix interphase promotes the formation of microcracks and additional transport pathways for moisture ingress [17,34]. These physicochemical and biological processes reinforce one another, creating a positive feedback mechanism in which increasing moisture promotes microbial activity, whereas biodeterioration further facilitates retained moisture within the composite.

The resulting microstructural changes substantially modify both heat and moisture transport. Gradual degradation of the pore system, together with increasing structural heterogeneity, enhances moisture migration and reduces the effectiveness of air-filled pores as thermal barriers. Consequently, thermal conductivity gradually increases, while the insulating performance of the material deteriorates [17,34,41]. This phenomenon is particularly important in building envelopes exposed to repeated wetting–drying cycles, where long-term environmental loading continuously accelerates both physical ageing and biological degradation.

The coupled transport of heat and moisture represents an additional factor governing the long-term thermal behaviour of bio-based insulation materials. In porous biocomposites, these transport processes occur simultaneously and remain strongly interdependent throughout service life. Moisture migration therefore cannot be considered independently of heat flow, making hygrothermal coupling one of the key mechanisms controlling the durability and thermal efficiency of natural insulation materials [42,74].

Heat transfer in moisture-laden biocomposites extends beyond simple conductive mechanisms. Water present in warmer regions of the material evaporates while absorbing latent heat, and the resulting vapour migrates through the interconnected pore structure towards cooler zones, where condensation releases the stored thermal energy. Repeated evaporation–condensation cycles increase the apparent thermal conductivity (λapp) and progressively reduce the insulation efficiency of the composite [36,42].

Recent quantitative studies confirm that moisture content may substantially affect the thermal performance of bio-based building composites. Authors in [75] investigated hemp-shive composites with a magnesium-based binder in the dry state and after conditioning at 50%, 75% and 90% RH. Depending on material density and conditioning state, thermal conductivity values ranged from approximately 0.105 to 0.159 W/(m·K) under dry conditions and reached approximately 0.157–0.241 W/(m·K) at high relative humidity. The results demonstrate that the thermal response cannot be interpreted independently of both moisture content and density, because these parameters jointly determine heat transfer through the porous structure. Although the investigated material employed a mineral rather than a polymeric matrix, the observed moisture sensitivity provides an important building-material reference for evaluating the thermal consequences of moisture accumulation in lignocellulosic composites [75].

The moisture–thermal conductivity relationship is not necessarily linear across the entire hygroscopic range. Ref. [74] reported no significant variation in thermal conductivity of hemp-shive insulation between equilibrium states corresponding to 0 and 50% RH, whereas the influence of moisture became more pronounced at higher moisture levels. This behaviour indicates that moderate hygroscopic moisture uptake may initially have a limited effect on heat transfer, while greater pore-water accumulation at high relative humidity produces a substantially stronger thermal penalty. Consequently, thermal-conductivity measurements performed only under dry or moderate-RH conditions may underestimate the deterioration of insulation performance under prolonged high-humidity exposure [74].

Recent results obtained for flax and hemp shives further demonstrate that moisture modifies not only the absolute value of thermal conductivity but also its relationship with material density. Kosiński et al. showed that thermal conductivity of damp lignocellulosic shives increased noticeably with densification, whereas dry materials exhibited a different density-dependent response. This finding confirms that moisture content, pore structure and bulk density act as coupled variables controlling heat transfer. Such interactions are particularly relevant to biodeterioration because biological degradation may alter both moisture retention and pore architecture, potentially changing thermal performance even when the material subsequently undergoes partial drying [76].

Taken together, these studies demonstrate that moisture-induced deterioration of thermal performance is governed by a coupled interaction between moisture content, density and pore structure rather than by moisture uptake alone [74,75]. At low to moderate relative humidity, changes in thermal conductivity may remain limited, whereas high-humidity conditioning can increase λ by several tens of percent [75]. This non-linear response is particularly important for building applications because short-term measurements under standard laboratory conditions may not represent the thermal behaviour of bio-based materials exposed to prolonged high humidity or intermittent wetting during service.

In contrast to the relatively extensive literature on moisture-induced mechanical degradation, quantitative data describing the evolution of thermal conductivity in natural fibre-reinforced polymer composites during prolonged environmental or biological exposure remain scarce. Recent reviews of natural fibre-reinforced polymer composites continue to identify moisture sensitivity as a major durability limitation, but most experimental studies quantify water uptake and the associated loss of mechanical properties rather than simultaneously monitoring thermal conductivity. This imbalance is particularly important for construction applications, where mechanical integrity and thermal performance must be maintained throughout the service life of the material [11].

A further distinction must be made between potentially reversible moisture-induced changes in thermal conductivity and permanent thermal-performance deterioration associated with microstructural or biological damage. An increase in λ measured while a material is wet primarily reflects the replacement of low-conductivity air in the pore system by water and changes in heat-transfer pathways [37,42,43,44]. By contrast, biodeterioration may irreversibly modify pore geometry, fibre integrity and the fibre–matrix interface [17,34,46]. Consequently, measurements performed only in the wet state cannot determine whether the observed thermal penalty persists after drying. Comparative durability studies should therefore report thermal conductivity both before exposure and after controlled reconditioning to a common moisture state.

The available evidence therefore indicates that mechanical and thermal degradation should not be evaluated independently. Moisture penetration may simultaneously promote fibre swelling, interfacial debonding and microcracking, resulting in mechanical deterioration, while increasing heat transfer through the pore system [15,34,40,42,43,44,61]. If prolonged moisture exposure additionally enables fungal colonisation, enzymatic degradation of lignocellulosic fibres may further modify pore connectivity and material density [17,46,48,51]. However, very few studies quantify these processes simultaneously. As a result, direct correlations between fungal activity, microstructural damage, mechanical-property loss and changes in thermal conductivity have not yet been established for most polymer-based construction biocomposites [17,60,77].

**Figure 3:**
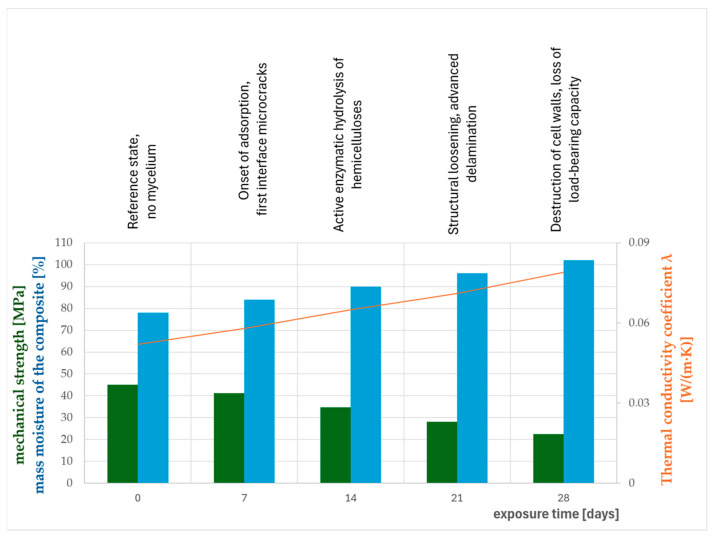
A conceptual model of changes in mechanical strength, mass moisture content, and thermal conductivity coefficient λ during the progressive biodeterioration of natural-fibre-reinforced biocomposites. Prepared by the author based on the literature [13,15,17,34,37,41,42].

Based on the mechanisms identified across the reviewed studies, thermal-performance deterioration can be conceptualised as a sequence initiated by moisture accumulation and potentially intensified when conditions become favourable for biodeterioration. During the early stages, fibre swelling and moisture uptake dominate the degradation process. Continued environmental and biological exposure may promote hydrolysis and enzymatic degradation of lignocellulosic constituents, weakening of the fibre–matrix interphase and progressive damage to fibre cell walls. As these processes advance, structural integrity may decline, while changes in pore structure and moisture retention can modify heat-transfer pathways. This conceptual relationship is illustrated in Figure 3.

Although the overall deterioration mechanism is well established, considerable variation exists in the magnitude of loss of thermal performance reported in the literature. Some studies attribute the increase in thermal conductivity primarily to retained moisture, considering changes in pore structure to be a secondary consequence of water uptake [36,37,41,42,43,44]. Other investigations indicate that fungal colonisation may induce persistent modifications of the internal pore network through degradation of lignocellulosic fibres and progressive development of microstructural defects [17,46]. Such irreversible microstructural changes could potentially affect thermal performance after drying; however, direct quantitative evidence confirming persistent changes in λ after fungal exposure and reconditioning remains limited.

These differences largely reflect variations in experimental methodology rather than contradictory deterioration mechanisms. Many investigations have focused on short-term moisture absorption under controlled laboratory conditions [42,43,44], whereas studies addressing biodeterioration generally involve prolonged fungal exposure under sustained high-humidity environments lasting several weeks [17,46,78]. In practical building applications, however, retained moisture and microbial activity rarely occur independently. Instead, they develop simultaneously and continuously interact throughout the service life of the material. Consequently, the deterioration of thermal insulation performance under real operating conditions may differ substantially from that predicted from laboratory studies considering moisture exposure alone [10,14,36].

**Figure 4:**
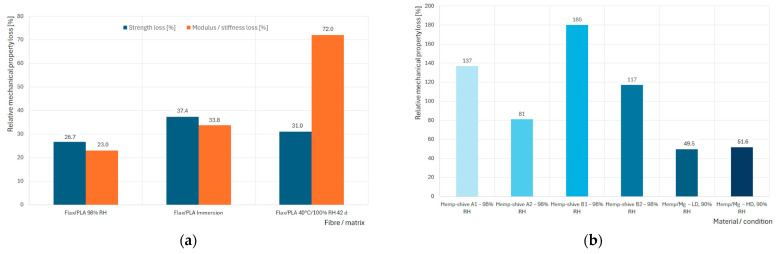
Quantitative comparison of degradation-related property changes reported in the literature: (**a**) reductions in selected mechanical properties of natural fibre-reinforced polymer composites following moisture, hygrothermal or biological exposure; (**b**) moisture-dependent increase in thermal conductivity of representative bio-based building materials. Values were compiled from published studies under different experimental conditions and are intended to illustrate the reported magnitude of change rather than direct material-to-material equivalence [63,64,74,75].

To enable direct quantitative comparison across different material systems and exposure conditions, the reported mechanical and thermal properties were normalised relative to the corresponding unaged or reference state. Figure 4 compares relative changes in mechanical properties following moisture, hygrothermal and biological exposure, with moisture-induced changes in thermal conductivity reported for bio-based insulation materials.

The thermal data reveal a strongly non-linear moisture response. While moderate humidity produced limited changes in some hemp-shive insulation systems, conditioning at 90–98% RH increased thermal conductivity by approximately 50% to as much as 180%, depending on material formulation and density. This wide range demonstrates that moisture sensitivity is highly material-specific and that dry-state thermal conductivity alone may substantially underestimate insulation losses under severe humid exposure.

The quantitative comparison reveals substantial differences in the magnitude of degradation among the analysed material systems. Mechanical stiffness may exhibit greater sensitivity to environmental ageing than ultimate strength in some composite systems, as demonstrated for hygrothermally aged flax/PLA composites [64]. Such an early reduction in stiffness may indicate deterioration of fibre–matrix load-transfer efficiency before more extensive loss of ultimate load-bearing capacity [40,64]. The comparison also highlights an important imbalance in the available literature: quantitative data on moisture-induced mechanical degradation are relatively abundant [40,63,64], whereas studies directly linking fungal colonisation with simultaneous changes in mechanical and thermal properties remain scarce [56,70].

Overall, the available literature demonstrates that moisture exerts a pronounced influence on the thermal performance of bio-based building materials [74,75]. However, directly comparable quantitative data describing changes in thermal conductivity of polymer-based natural-fibre composites during fungal biodeterioration remain scarce. Consequently, although the physical mechanisms responsible for moisture-related changes in thermal performance are relatively well understood [74,75], their quantitative relationship with active biological degradation has not yet been sufficiently established. This limitation highlights the need for integrated experimental programmes combining controlled biological exposure, continuous moisture monitoring, thermal-conductivity measurements and microstructural characterisation under conditions representative of building-service environments.

**Figure 5:**
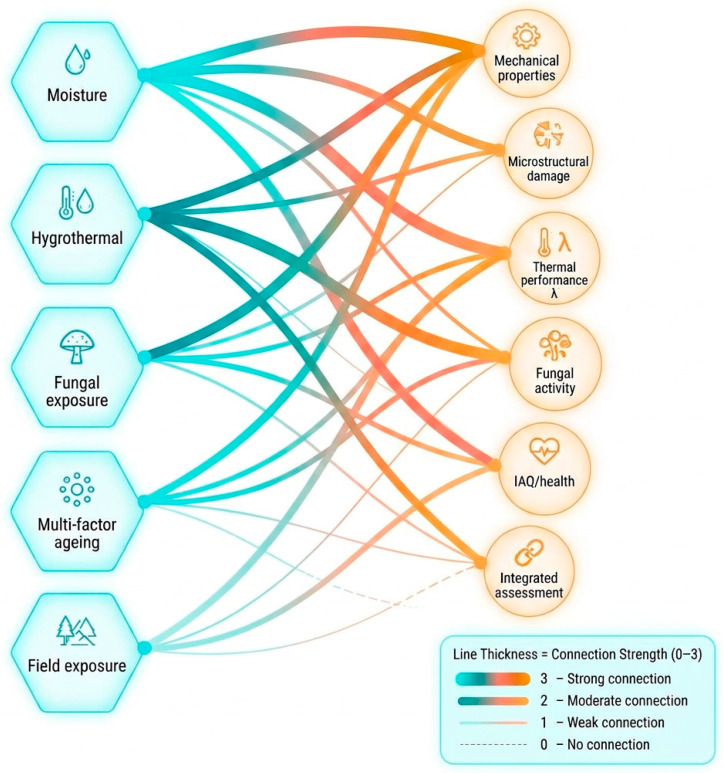
Semi-quantitative evidence map linking the main exposure scenarios with mechanical, microstructural, thermal, biological and indoor-environmental outcomes reported for natural fibre-based construction biocomposites. Line thickness represents the relative strength of the available evidence (3—strong, 2—moderate, 1—limited, 0—no or insufficient direct evidence) based on the critical synthesis of the reviewed literature rather than a formal bibliometric count or the magnitude of a physical causal effect.

The reviewed literature shows a pronounced imbalance in the availability and integration of evidence across different exposure scenarios and performance indicators. Moisture and hygrothermal ageing are relatively well documented in relation to mechanical deterioration and thermal performance [40,63,64,74,75], whereas direct quantitative links between fungal exposure and changes in engineering performance remain less established [56,69,70,79]. Field-scale investigations are also comparatively scarce, particularly for polymer-based natural fibre composites under realistic building-service conditions. To visualise these differences, Figure 5 presents a semi-quantitative evidence map linking the principal exposure scenarios with the corresponding material-performance and biological outcomes. Connection strength reflects the relative availability, consistency and comparability of evidence identified in the reviewed literature rather than the magnitude of a physical cause-and-effect relationship.

As illustrated in Figure 5, the strongest evidence concerns the effects of moisture and hygrothermal exposure on mechanical properties and microstructural deterioration. Quantitative thermal data are also available for moisture-sensitive bio-based insulation systems, although substantially fewer studies address thermal-performance changes under controlled fungal exposure. Fungal activity itself is well documented under microbiological testing conditions, but direct correlations with residual mechanical properties, thermal conductivity or indoor-air-quality indicators remain limited. The weakest evidence concerns integrated assessments combining environmental exposure, microbial activity, microstructural evolution and functional-property loss within the same experimental programme, particularly under field or in-service conditions. This imbalance identifies integrated and field-validated durability assessment as one of the principal research gaps in the current literature.

### 4.3. Impact of Biodeterioration on Indoor Air Quality and Potential Health Risks

The consequences of biodeterioration extend beyond the deterioration of material properties and may also affect indoor environmental quality when fungal colonisation occurs on materials used within the building envelope or interior environment [79]. Colonised materials may act as sources of airborne fungal components and metabolic products, including spores, hyphal fragments, microbial volatile organic compounds (MVOCs) and, depending on the fungal species and environmental conditions, mycotoxins [80].

Indoor air quality (IAQ) is therefore closely linked to the biological durability of bio-based construction materials. As fungal colonies develop within porous insulation systems, increasing quantities of biological contaminants may be emitted into occupied spaces. Exposure to damp and mould-contaminated indoor environments has been associated with increased risks of respiratory and asthma-related health outcomes, including respiratory symptoms, wheeze, cough and asthma exacerbation [81]. Other indoor exposure indicators associated with fungal contamination and dampness have also been linked to oculo-nasal and related symptoms [79,82].

The likelihood of microbial contamination depends not only on the extent of fungal growth but also on environmental conditions that promote prolonged retained moisture within building envelopes. Elevated humidity accelerates fungal development, while the porous microstructure of natural insulation materials provides an extensive surface area that facilitates colonisation. As biodeterioration progresses, both the concentration and diversity of airborne biological contaminants may increase, further compromising indoor air quality and potentially affecting occupant health [14,83].

Quantitative field studies confirm that moisture-damaged building environments can be associated with substantial airborne fungal loads, although reported concentrations vary widely depending on building condition, season, fungal species and sampling methodology. Investigations of moisture- and flood-affected buildings have reported airborne fungal concentrations ranging from low or non-detectable levels to more than 10^3^ CFU/m^3^, with individual measurements reaching approximately 2.4 × 10^3^ CFU/m^3^. Dust reservoirs may contain considerably higher fungal loads, reaching approximately 2.5 × 10^5^ CFU/g in contaminated environments. These results demonstrate that fungal contamination cannot be characterised solely by the visible extent of surface growth because substantial microbial reservoirs may occur within porous materials and settled dust and may contribute intermittently to airborne exposure [84].

MVOCs provide an additional potential indicator of microbial activity, but their interpretation is more complex than direct enumeration of fungal propagules. Compounds commonly classified as MVOCs may originate from both microbial and non-microbial indoor sources, and their indoor concentrations can additionally be influenced by environmental conditions. Consequently, individual MVOCs should not be interpreted as universal quantitative markers of fungal biomass or material degradation without supporting microbiological evidence [85].

Recent epidemiological evidence further demonstrates that specific volatile compounds associated with damp or microbially affected indoor environments may correlate with occupant symptoms. In a 2024 study involving 159 adults from homes in three Nordic cities, higher levels of selected volatile compounds, including 3-methylfuran, 2-hexanone, 2-heptanone and 1-octen-3-ol, were significantly associated with oculo-nasal symptoms, while higher total mould levels were also associated with increased symptom prevalence. For example, associations with oculo-nasal symptoms were reported for 3-methylfuran (*p* = 0.003), 2-hexanone (*p* = 0.037), 2-heptanone (*p* = 0.018) and 1-octen-3-ol (*p* = 0.001). These findings provide quantitative evidence linking microbial or dampness-related indoor exposure indicators with occupant health outcomes, although they do not establish a direct relationship with the degree of mechanical or thermal degradation of the underlying construction material [79].

Recent analytical developments also demonstrate the potential of volatile-compound profiling for detecting fungal contamination before extensive visible deterioration occurs. Choi et al. combined SPME–GC–MS analysis with machine-learning classification to distinguish fungal-associated VOC profiles from emissions originating from common indoor materials. A random-forest model achieved a classification accuracy of 96.2%, while compounds including ethanol, 2-butanone and other volatile markers contributed to discrimination between fungal and non-fungal emission profiles. These results indicate that VOC profiling may complement conventional visual and microbiological inspection, although such approaches currently identify fungal contamination rather than quantify the associated loss of engineering performance [86].

Despite these quantitative advances, a direct dose–response relationship between fungal burden, MVOC emission and deterioration of mechanical or thermal properties has not yet been established for natural fibre-reinforced polymer biocomposites used in construction. Existing studies generally investigate these endpoints separately: indoor-air studies quantify airborne fungi, fungal markers or volatile metabolites [79,84,85,86], whereas material-durability studies report changes in strength, stiffness, moisture behaviour or thermal conductivity [56,63,64,75]. Because these parameters are rarely measured simultaneously in the same material system under identical exposure conditions, combining values from independent studies into a single statistical correlation would not be methodologically justified. The available evidence therefore supports a conceptual chain linking moisture accumulation, fungal activity, contaminant emission and material deterioration, but the quantitative coupling between biological exposure indicators and engineering performance remains an important research gap.

The available quantitative evidence linking fungal contamination, indoor exposure indicators and material-performance deterioration is summarised in Table 7.

**Figure 6:**
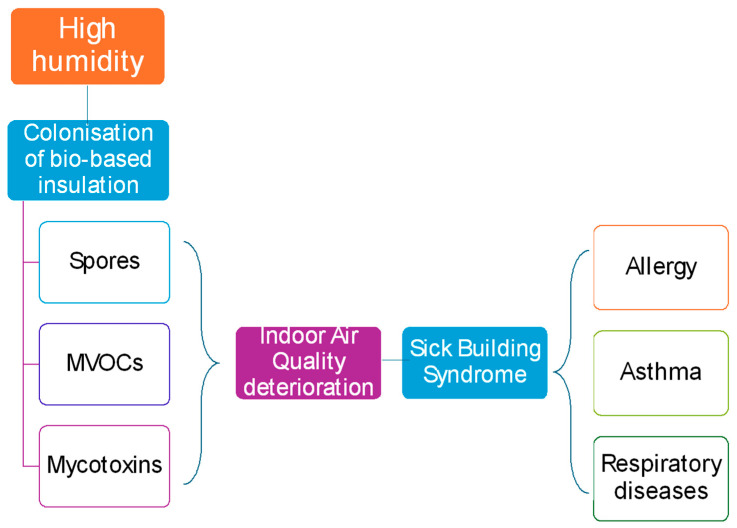
Conceptual model illustrating the influence of moisture-induced fungal colonisation of bio-based building materials on indoor air quality (IAQ). Prepared by the author based on the literature [14,81,82].

The conceptual relationship between retained moisture, fungal colonisation and indoor air quality is illustrated in Figure 6. Prolonged moisture exposure initiates microbial growth, which subsequently leads to the release of fungal spores, microbial volatile organic compounds (MVOCs) and, in some cases, mycotoxins. These emissions contribute to deterioration of indoor environmental quality and may increase the risk of respiratory disorders, allergic reactions and symptoms associated with Sick Building Syndrome [14,81,82].

The relationship between biodeterioration and indoor environmental quality has received increasing attention in recent years, particularly in the context of sustainable construction [79]. Although natural fibre-reinforced materials provide significant environmental benefits, their long-term performance should be evaluated not only in terms of mechanical durability and thermal efficiency but also with respect to their influence on indoor environmental quality and occupant health. Biological durability should therefore be regarded as an integral component of sustainability assessment rather than as a separate material characteristic.

Current evidence consistently indicates that effective moisture management remains the most effective strategy for limiting microbial colonisation and preserving indoor air quality [14,82,83]. Appropriate building design, efficient ventilation and effective vapour control reduce the likelihood of prolonged retained moisture and, consequently, minimise the risk of fungal growth and the release of biologically active contaminants. Preventive measures implemented during the design and operation of buildings are therefore considerably more effective and environmentally sustainable than remediation following microbial contamination.

From an engineering perspective, assessment of construction biocomposites should extend beyond conventional evaluation of mechanical strength and thermal performance. Future durability studies should integrate analyses of microbial colonisation, indoor air quality indicators and potential health risks throughout the service life of bio-based materials. Such an integrated approach would provide a more realistic assessment of long-term performance under operating conditions and support the development of construction systems that are not only durable and energy-efficient but also safe for building occupants.

Overall, the available evidence demonstrates that biodeterioration simultaneously affects the structural, thermal and environmental performance of natural fibre-reinforced biocomposites. Moisture acts as the initiating factor, whereas microbial activity accelerates degradation of the material and amplifies its consequences for both engineering performance and indoor environmental quality. These interactions highlight the need for integrated durability assessment that combines mechanical, hygrothermal and biological analyses.

## 5. Eco-Friendly Mitigation Strategies

Service durability remains one of the principal challenges limiting the wider application of natural fibre-reinforced biocomposites in construction. As discussed in the preceding sections, biodeterioration is initiated primarily by retained moisture and subsequently accelerated by microbial activity. Consequently, improving biological durability requires strategies capable of simultaneously restricting moisture ingress, stabilising the fibre–matrix interphase and limiting microbial colonisation without compromising the environmental advantages of bio-based materials.

Current research has therefore shifted from conventional preservation methods based on synthetic biocides towards more environmentally sustainable solutions. Particular attention has been devoted to physical and chemical modification techniques that enhance durability while preserving recyclability, biodegradability and overall environmental compatibility. These approaches are closely aligned with the principles of green chemistry and support the broader objectives of sustainable construction and circular material use [13,14,87].

**Figure 7:**
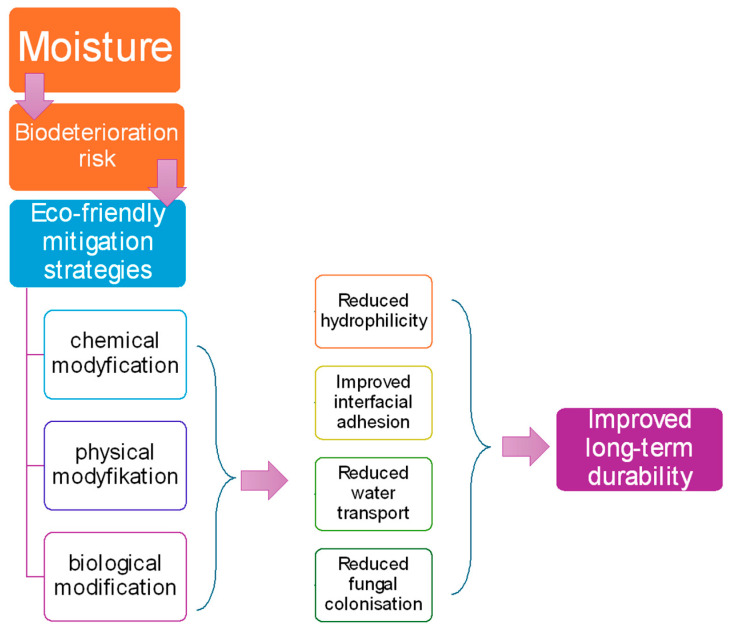
Methods for improving the biological durability of natural fibre-reinforced biocomposites by reducing moisture absorption, enhancing fibre-matrix bonding, and limiting microorganism growth. Prepared by the author based on the literature [13,14,87,88].

The most widely investigated mitigation strategies include chemical modification of lignocellulosic fibres, optimisation of polymer matrices and the application of environmentally friendly surface treatments. Although these approaches differ in their mechanisms of action, they pursue a common objective: reducing moisture transport within the composite, improving the stability of the fibre–matrix interphase and suppressing conditions favourable for microbial growth. Their relationships and complementary roles in enhancing the biological durability of natural fibre-reinforced biocomposites are summarised schematically in Figure 7.

### 5.1. Chemical and Physical Modifications

Chemical and physical modification of lignocellulosic fibres is widely regarded as one of the most effective approaches to improving the service durability of natural fibre-reinforced biocomposites. Because biodeterioration is initiated primarily by moisture uptake, most modification techniques are designed to reduce fibre hygroscopicity, enhance compatibility with hydrophobic polymer matrices and increase resistance to biological and environmental degradation [16,89].

A common objective of these treatments is to reduce the number of accessible hydroxyl groups on the fibre surface, thereby limiting water absorption and improving fibre–matrix adhesion. Enhanced interfacial bonding restricts the formation of interfacial voids and microdefects that otherwise facilitate moisture transport and provide favourable sites for microbial colonisation [90]. Consequently, improved interfacial stability contributes not only to enhanced mechanical performance but also to greater biological durability during long-term service.

Recent research has increasingly focused on hybrid modification strategies that combine conventional chemical treatments with mineralisation processes or nanostructured surface modifications [89,90]. These integrated approaches simultaneously improve several durability-related characteristics by strengthening the fibre–matrix interphase, reducing moisture transport and providing additional protection against microbial attack. Their multifunctional nature makes them particularly attractive for the development of next-generation sustainable construction biocomposites.

**Figure 8:**
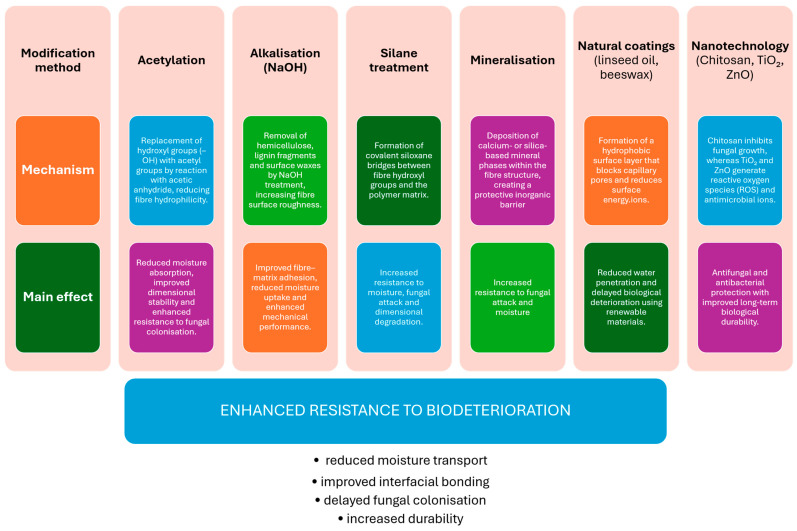
Eco-friendly modification strategies for improving the biological durability of natural fibre-reinforced biocomposites. Prepared by the author based on the literature [16,77,89,90,91,92,93].

The principal environmentally friendly fibre modification techniques, together with their mechanisms of action and their contribution to enhanced biological durability, are summarised schematically in Figure 8.

### 5.2. Natural Coatings and Nanotechnology

The development of environmentally friendly protective systems has become one of the most dynamic research directions in the field of natural fibre-reinforced biocomposites. Rather than relying on conventional synthetic biocides, current strategies increasingly utilise renewable materials and functional nanomaterials that improve biological durability while preserving the environmental advantages of bio-based composites [91]. Most available approaches aim either to reduce moisture availability or to suppress microbial activity without adversely affecting the recyclability or biodegradability of the material. The principal eco-friendly protection strategies include:

- natural oils and waxes—These treatments create a hydrophobic barrier on the fibre surface, reducing capillary water uptake and limiting moisture penetration into the lignocellulosic structure. By decreasing water availability, they indirectly inhibit fungal colonisation while preserving the environmental compatibility of the composite [91];
- chitosan—This naturally derived biopolymer exhibits well-documented antifungal activity. Its protective action is primarily associated with disruption of fungal cell membranes and cell walls, thereby inhibiting microbial growth and reducing colonisation of lignocellulosic fibres. Because chitosan is biodegradable, renewable and non-toxic, it is considered one of the most promising bio-based protective coatings for sustainable construction materials [77];
- photocatalytic nanoparticles (TiO_2_ and ZnO)—These nanomaterials provide active antimicrobial protection through the generation of reactive oxygen species (ROS) under appropriate irradiation. The resulting oxidative stress damages microbial cells and promotes degradation of organic contaminants. In addition to their photocatalytic activity, ZnO nanoparticles release Zn^2+^ ions, providing supplementary antibacterial and antifungal effects [92,93].

Linseed oil treatment provides a representative example of hydrophobic protection. Rather than directly eliminating microorganisms, it modifies the surface properties of the composite by reducing surface energy and blocking capillary pores, thereby restricting moisture transport into the material. In contrast to conventional biocides, its protective effect results primarily from limiting the environmental conditions required for microbial growth. Nevertheless, natural coatings gradually lose effectiveness because of oxidation, weathering and leaching, making them most suitable as components of multilayer protective systems rather than as standalone solutions [16,42,91].

Comparison of currently available protection strategies indicates that no individual treatment completely prevents biodeterioration. Chemical modification methods, including acetylation and alkaline treatment, effectively reduce moisture sorption and improve fibre–matrix adhesion but cannot entirely eliminate microbial colonisation during prolonged exposure to humid environments. Likewise, natural hydrophobic coatings provide effective moisture protection, although their performance gradually declines as the material ages under environmental exposure [16,77,89,90,91,92,93].

Current research therefore increasingly favours hybrid protection systems that combine complementary mechanisms of action. Integrating fibre surface modification with biologically active coatings enables simultaneous reduction of moisture transport, improvement of fibre–matrix compatibility and inhibition of microbial growth. By addressing several degradation mechanisms at the same time, these multifunctional systems offer greater potential for improving service durability than individual treatments applied separately.

Despite these encouraging developments, the available evidence remains dominated by relatively short-term laboratory investigations, most of which do not exceed several months of exposure. Consequently, knowledge of the long-term performance of eco-friendly protection systems under realistic building operating conditions is still limited. Future studies should therefore focus on extended environmental exposure, field validation and service durability monitoring before these technologies can be confidently implemented on a wider scale in sustainable construction [14].

### 5.3. Hybrid Anti-Biodeterioration Strategies

Although individual fibre treatments and protective coatings can improve selected durability characteristics, no single modification strategy simultaneously addresses all mechanisms governing biodeterioration of natural fibre-reinforced polymer biocomposites. Alkali treatment can modify fibre surface characteristics and improve interfacial adhesion, although excessive treatment may damage the lignocellulosic structure; silane coupling agents can improve fibre–matrix compatibility and reduce fibre hydrophilicity, but these treatments are not inherently intended to provide long-term antimicrobial protection [90]. Antimicrobial or photocatalytic nanoparticles can suppress microbial growth, but their presence alone does not necessarily eliminate moisture transport through the composite. These complementary functions provide a rationale for developing hybrid protection strategies that combine interfacial modification, moisture control and biological protection.

The principal advantage of hybrid modification lies in the possibility of acting at different stages of the biodeterioration pathway. Fibre pretreatment can reduce hydrophilicity and improve interfacial compatibility, coupling agents can improve bonding at the fibre–matrix interface, while antimicrobial or photocatalytic components can inhibit microbial colonisation [90,94]. Combined alkali–silane treatment has been experimentally demonstrated as an effective approach for modifying natural-fibre surface and interfacial characteristics [90], whereas other multifunctional combinations involving antimicrobial or photocatalytic components represent promising strategies whose effectiveness depends strongly on material composition, treatment concentration, processing conditions and exposure environment. Synergistic effects should therefore not be assumed unless demonstrated experimentally. A bioactive propolis–silane system represents a directly investigated example of a hybrid antifungal strategy for lignocellulosic–polymer composites. This approach combines silane-based interfacial modification with the biological activity of propolis and has been experimentally evaluated for fungal resistance in wood-filled polypropylene composites [94].

Experimentally demonstrated and emerging hybrid or multifunctional protection strategies relevant to natural fibre-based construction biocomposites are compared in Table 8. The level of evidence differs substantially between individual approaches; therefore, systems directly validated in natural fibre/polymer composites are distinguished from multifunctional concepts demonstrated primarily in related coatings or bio-based substrates.

Among the available approaches, alkali–silane modification primarily addresses interfacial durability rather than biological activity itself. Its principal benefit arises from improved fibre–matrix adhesion and reduced preferential moisture transport, thereby indirectly limiting conditions favourable to microbial development. By contrast, systems incorporating TiO_2_, ZnO or other antimicrobial agents introduce an additional active mechanism capable of suppressing microbial colonisation. Hybrid systems combining moisture control with antimicrobial activity are therefore conceptually more comprehensive because they target both the initiating conditions for biodeterioration and subsequent biological growth [94].

The suitability of individual hybrid systems is nevertheless application-dependent. Photocatalytic TiO_2_-containing systems are particularly attractive for externally exposed components receiving sufficient irradiation, whereas their photocatalytic effectiveness may be reduced in dark wall cavities or internal insulation layers [103,104]. In such environments, non-photocatalytic antimicrobial systems or moisture-control strategies may be more appropriate. Similarly, structural composites require modification strategies that preserve fibre strength and fibre–matrix load transfer [90,95,96], whereas surface-oriented protection may be more suitable for non-load-bearing panels in which biodeterioration is predominantly initiated at the exposed surface. Therefore, the selection of a protection system should be based on the expected exposure scenario and functional role of the building component rather than solely on laboratory antifungal efficacy.

Despite promising laboratory results, evidence for the long-term superiority of hybrid systems over single-component treatments remains limited. Most available studies evaluate selected endpoints, such as water absorption and mechanical or interfacial properties [90,96,101,102], or photocatalytic and antimicrobial performance [97,98,99,103,104], whereas relatively few investigate the simultaneous retention of interfacial integrity, antifungal performance and functional properties during prolonged cyclic ageing. Potential concerns include nanoparticle stability and release, coating degradation, compatibility with recycling processes and the additional environmental burdens that may arise from multistage chemical treatments. Future studies should therefore compare hybrid and single-component treatments using identical exposure protocols and incorporate long-term durability, recyclability and life-cycle assessment into the evaluation of protective efficiency.

Recent LCA-oriented studies further demonstrate that durability-related improvements should not be evaluated independently of the environmental burdens associated with fibre treatment. Yadav et al. showed that a conventional chemical-treatment route resulted in an approximately 87% higher environmental impact than the investigated natural-treatment alternative, despite providing improvements in mechanical performance and substantial reductions in water absorption. This trade-off illustrates that a treatment offering favourable technical performance does not necessarily provide the lowest life-cycle environmental impact [105].

## 6. Test Methods for Assessing Biodeterioration and Gaps in Standards

Reliable assessment of the service durability of natural fibre-reinforced biocomposites remains one of the major methodological challenges in contemporary materials engineering. Although several standardised procedures are available for evaluating fungal resistance, most were originally developed for conventional polymeric materials rather than porous lignocellulosic composites used in construction. Consequently, their applicability to bio-based building materials is limited [12].

The ISO 846 (Evaluation of the action of microorganisms on plastics) provides standardised procedures for assessing the susceptibility of plastics to deterioration caused by fungi, bacteria and soil microorganisms [60]. However, its direct applicability to highly porous and hygroscopic lignocellulosic composites is limited because their moisture transport, internal colonisation and degradation mechanisms differ substantially from those of conventional polymeric materials [12]. Related standardised methods include ASTM G21, which evaluates fungal resistance of synthetic polymeric materials, and ASTM D3273, which assesses resistance to mould growth on the surface of interior coatings in an environmental chamber [106,107]. Although useful for comparative screening, these procedures do not fully reproduce the coupled moisture transport, internal colonisation and property deterioration characteristic of porous natural fibre-based construction composites.

These limitations become particularly evident in the case of lignocellulosic construction biocomposites. Owing to their highly porous three-dimensional structure, conventional agar-based Petri dish tests frequently provide an incomplete representation of degradation processes, leading to either underestimation or overestimation of biodeterioration [12]. Standard procedures primarily assess visible fungal growth on the material surface while overlooking fungal penetration into the pore network, internal moisture redistribution and progressive changes in material properties resulting from repeated wetting–drying cycles [12,46].

The available literature therefore increasingly indicates that conventional microbiological testing should be complemented by advanced experimental techniques capable of reproducing in-use conditions and monitoring degradation throughout the entire material volume rather than only at its surface [12]. The most promising complementary methods include:

- Dynamic hygrothermal chamber testing—Enables controlled simulation of cyclic temperature and relative humidity conditions representative of real building operation. This approach allows simultaneous evaluation of retained moisture, fungal colonisation and the service durability of construction biocomposites under realistic environmental loading [83].
- Acoustic emission (AE)—Provides continuous, non-destructive monitoring of active damage processes by recording transient elastic waves generated during microcracking, interfacial debonding, fibre-related damage and other structural changes. AE has been successfully applied to characterise damage mechanisms in natural fibre-reinforced polymer composites and to assess changes in damage evolution following hygrothermal ageing [108,109]. When combined with controlled biological exposure, AE could therefore support temporal monitoring of damage development, although complementary microbiological and microstructural methods are required to determine its specific biological or physicochemical origin.
- X-ray micro-computed tomography (µCT)—Enables three-dimensional, non-destructive characterisation of the internal microstructure. Quantitative analysis of pore evolution, crack propagation, fibre architecture and internal structural damage can be performed without destroying the specimen. When combined with microbiological or imaging techniques, µCT may additionally help relate internal structural changes to the spatial progression of biodeterioration [110].

**Figure 9:**
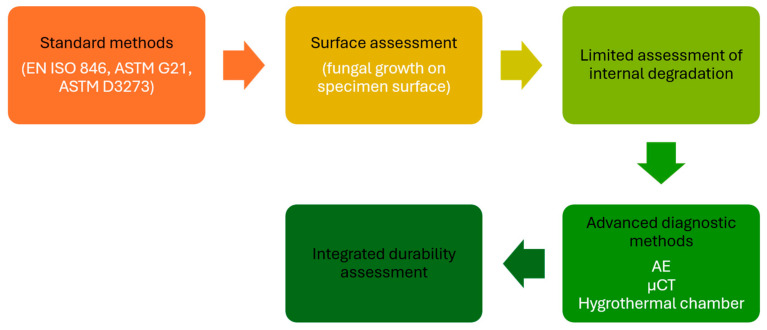
Conceptual framework illustrating the integration of conventional standardised methods and advanced diagnostic techniques for assessing biodeterioration of bio-based building materials. Prepared by the author based on the literature [12,46,78,91,106,107].

The complementary roles of conventional standardised procedures and advanced diagnostic techniques are illustrated schematically in Figure 9.

Although ISO 846:2019 provides established procedures for evaluating the action of microorganisms on plastics, its applicability to highly porous and hygroscopic natural fibre-reinforced construction biocomposites remains limited. Under practical service conditions, biodeterioration is governed not only by microbial growth but also by cyclic fluctuations in temperature and relative humidity, moisture retention, vapour transport, pore connectivity and the persistence of localised wet zones. These interacting mechanisms are only partially represented in conventional laboratory procedures [12,46,60,78]. Consequently, harmonised protocols integrating controlled biological exposure with subsequent mechanical, thermal and microstructural assessment are still required for construction biocomposites. For this reason, increasing attention is being directed towards combined testing methodologies that combine standardised microbiological procedures with advanced non-destructive diagnostic techniques and continuous monitoring of hygrothermal parameters [12,46,78,79]. Such integrated approaches provide a more realistic assessment of degradation processes occurring throughout the service life of bio-based building materials and allow stronger links to be established between microstructural evolution and changes in engineering performance.

**Figure 10:**
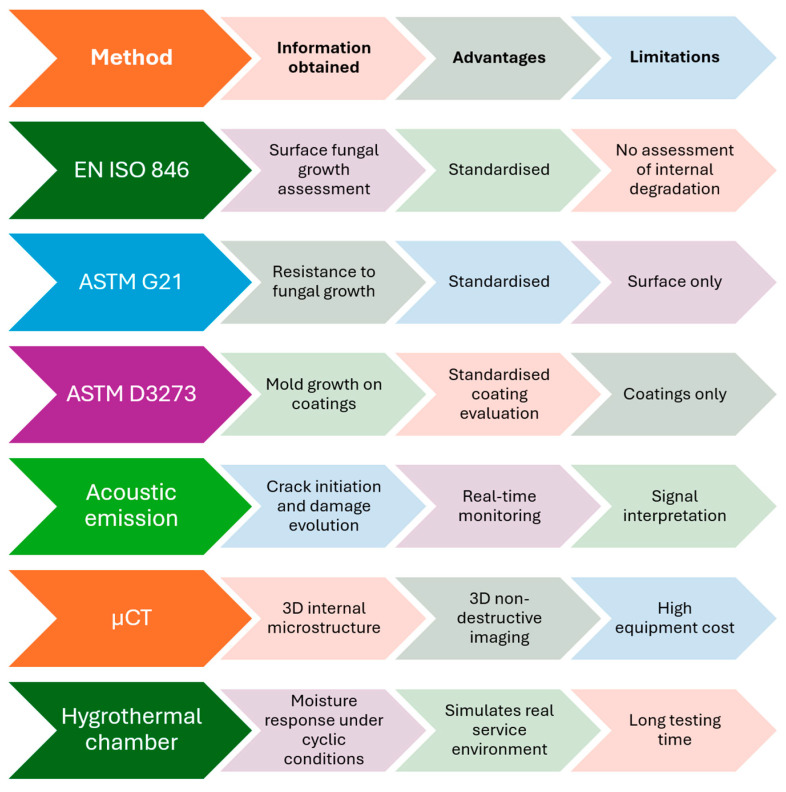
Comparison of conventional standardised methods and advanced diagnostic techniques used for assessing the biodeterioration of bio-based building materials. Prepared by the author based on the literature [12,46,78,91,106,107].

The currently available methods also differ substantially in both the type and the level of information they provide. Conventional standardised tests primarily assess fungal growth or material susceptibility under prescribed exposure conditions, whereas complementary diagnostic techniques can provide additional information on internal structural degradation, moisture redistribution and damage development within the fibre–matrix system. Their complementary capabilities are summarised in Figure 10.

As illustrated in Figure 10, no single experimental method currently provides a comprehensive assessment of biodeterioration. Standardised microbiological tests remain essential for evaluating fungal resistance, whereas advanced diagnostic techniques reveal the evolution of internal damage that cannot be detected using conventional procedures alone. Future assessment of natural fibre-reinforced construction biocomposites should therefore be based on integrated experimental frameworks combining microbiological, hygrothermal and microstructural analyses. Such an approach offers the most reliable basis for predicting service durability under in-use conditions.

## 7. Current Research Challenges and Directions for Further Research

Despite the rapid development of natural fibre-reinforced polymer biocomposites, several scientific and technological challenges continue to limit their broader application in construction. The available literature demonstrates substantial progress in understanding individual aspects of biodeterioration and material performance. Nevertheless, current knowledge remains fragmented, as most studies focus on isolated degradation mechanisms rather than on the complex interactions governing service durability under in-use conditions.

The analysis of the reviewed literature identifies several priority areas requiring further investigation:

- Fragmented durability assessment—Most published studies evaluate mechanical performance, moisture behaviour or biological resistance separately. Under actual service conditions, however, these processes are strongly interrelated. Retained moisture promotes fungal colonisation, microbial activity modifies the internal microstructure, while increasing porosity accelerates both moisture transport and thermal conductivity. Future studies should therefore adopt integrated experimental approaches capable of assessing these coupled degradation mechanisms simultaneously [6,17].
- Lack of harmonised testing procedures—Considerable differences in fungal species, exposure duration, environmental conditions and experimental methodologies limit direct comparison between published investigations. The development of harmonised testing protocols would significantly improve the reliability, reproducibility and comparability of durability data generated by different research groups.
- Limited representation of real service conditions—Most experimental investigations have been conducted under constant laboratory conditions, whereas construction materials are exposed to continuously changing temperature, relative humidity and moisture regimes throughout their service life. Future experimental programmes should therefore incorporate cyclic hygrothermal loading that more realistically reproduces environmental conditions encountered in buildings [16,33,60].
- Insufficient understanding of coupled degradation mechanisms—Moisture transport, thermal performance and biodeterioration continue to be investigated largely as independent processes despite their strong interdependence. Moisture accelerates microbial activity, whereas degradation of lignocellulosic fibres modifies pore structure and further enhances both moisture migration and heat transfer. Quantitative descriptions integrating these interacting mechanisms remain scarce [15,37,42].
- Limited service durability data—Most available studies cover exposure periods ranging from several weeks to only a few months. Long-term investigations extending over several years and incorporating seasonal climatic variations and natural weathering remain rare, despite their importance for predicting the service life of building materials intended to operate for decades [6,39,41,54].
- Need for multifunctional protection systems—Hybrid strategies combining fibre modification, hydrophobic coatings and antimicrobial nanomaterials represent one of the most promising directions for future research because they simultaneously reduce moisture uptake, improve fibre–matrix compatibility and inhibit microbial growth. Nevertheless, their service durability, environmental impact and influence on recyclability remain insufficiently documented.
- Integrated durability assessment and predictive modelling—Future investigations should increasingly combine long-term environmental exposure, advanced non-destructive diagnostics, microstructural characterisation and predictive numerical modelling. Such integrated methodologies would facilitate quantitative relationships between microstructural evolution and changes in mechanical and thermal performance, ultimately enabling more reliable prediction of service life.

Service-life prediction approaches can be broadly classified into empirical, mechanistic, coupled hygrothermal–biological and data-driven models. Empirical models extrapolate degradation trends obtained from accelerated ageing or environmental exposure tests and are relatively simple to implement, but their predictive reliability is limited when exposure conditions differ from those used for calibration. Mechanistic models describe individual deterioration processes, such as moisture diffusion, fibre swelling, interfacial damage or strength loss, and therefore provide greater physical interpretability; however, they require material-specific parameters that remain poorly characterised for natural fibre-reinforced biocomposites. Coupled hygrothermal–biological approaches provide a more realistic framework by linking transient temperature and moisture conditions with mould-growth risk, as demonstrated by established mould-growth models and their application to dynamic hygrothermal simulations of building envelopes [111,112]. However, these approaches primarily predict conditions favourable to microbial development or mould-growth indices rather than the resulting loss of mechanical or thermal performance. More recently, machine-learning approaches have created opportunities to analyse multidimensional datasets describing composite composition, processing conditions, environmental exposure and resulting material performance. Recent reviews demonstrate rapidly growing applications of machine learning to natural fibre composites and fibre-reinforced polymers, including property prediction, material optimisation and durability assessment [113,114]. However, model reliability and generalisability remain strongly dependent on the quantity, quality and representativeness of the available training data, while long-term degradation datasets remain particularly limited for natural fibre-reinforced construction biocomposites [115,116].

Digital-twin approaches represent a further development of predictive durability assessment rather than a separate degradation model [117,118]. In the built environment, digital twins increasingly integrate sensor-based monitoring, BIM/asset information, computational models and data-driven analytics to support dynamic condition assessment, predictive maintenance and lifecycle management [117,118]. At the material level, emerging multiscale digital-twin concepts additionally aim to represent microstructure-dependent behaviour and progressive damage, providing a potential framework for continuously updating material-condition predictions as new monitoring or experimental data become available [119]. For natural fibre-reinforced construction biocomposites, such an approach could potentially integrate measured temperature and relative-humidity histories, moisture accumulation, conditions favourable to fungal growth and non-destructive diagnostic data with models describing progressive changes in mechanical or thermal performance. However, practical implementation remains at an early stage because validated relationships linking environmental exposure, microbial activity, microstructural deterioration and functional-property loss are still lacking. Consequently, digital twins should currently be regarded as a promising future framework for integrating monitoring and prediction rather than as an established method for service-life assessment of biodeteriorating biocomposites.

**Figure 11:**
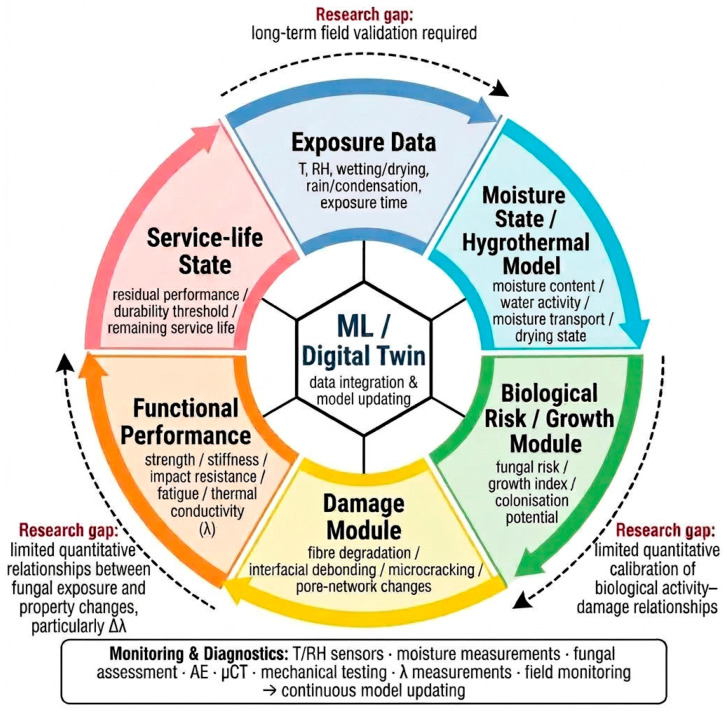
Proposed conceptual framework for integrated service-life prediction of natural fibre-reinforced construction biocomposites. The framework links environmental exposure and hygrothermal state with biological risk, microstructural damage, functional performance and remaining service life. Monitoring and diagnostic techniques provide complementary data for continuous model updating, while machine-learning and digital-twin approaches represent potential advanced tools for data integration and predictive assessment. Dashed arrows indicate major research gaps rather than established causal pathways. Prepared by the author based on the reviewed literature.

Based on the identified limitations, an integrated service-life assessment framework for natural fibre-reinforced construction biocomposites should link environmental exposure and hygrothermal conditions with biological activity, microstructural deterioration and changes in engineering performance. As conceptualised in Figure 11, monitoring and diagnostic data could support continuous model updating, while machine-learning and digital-twin approaches may provide an advanced integration layer for linking environmental histories with material-condition data and service-life predictions. However, three critical gaps currently limit implementation of such a framework: insufficient quantitative calibration between biological activity and damage development, limited quantitative relationships between fungal exposure and changes in engineering properties—particularly thermal conductivity—and the lack of long-term field validation.

The framework highlights that the principal limitation is not the absence of models or diagnostic methods for individual degradation processes, but the lack of validated quantitative links between successive stages of deterioration. Existing hygrothermal–biological models can estimate conditions favourable to fungal development, but these predictions cannot yet be translated reliably into corresponding changes in mechanical or thermal performance. In particular, quantitative relationships between fungal exposure and changes in thermal conductivity remain poorly established. Closing these gaps through coordinated laboratory testing, advanced diagnostics and long-term field monitoring is therefore essential for developing reliable service-life prediction tools.

The principal research priorities identified from the reviewed literature are summarised in Table 9.

The analysis presented in Table 9 demonstrates that future research should move beyond isolated laboratory investigations towards integrated durability assessment frameworks capable of reproducing realistic operating conditions. Combining long-term environmental exposure with advanced non-destructive diagnostic techniques, microstructural characterisation and predictive modelling will enable a more comprehensive understanding of degradation processes throughout the service life of construction biocomposites. Particular attention should be devoted to the development of harmonised testing methodologies and validated service-life prediction models linking changes in microstructure with the evolution of mechanical, thermal and biological performance. Such multidisciplinary approaches will be essential for the wider implementation of natural fibre-reinforced biocomposites in sustainable construction.

Accordingly, future development of natural-fibre biocomposites should move beyond the optimisation of initial mechanical properties towards integrated material-design strategies that simultaneously consider long-term durability, sustainable processing, circularity, end-of-life pathways and life-cycle environmental performance [20,120].

However, the technical effectiveness of such modification strategies should not be considered independently of their environmental consequences. Improvements in moisture resistance, interfacial stability or antimicrobial performance may require additional chemicals, energy-intensive processing or components that complicate recycling and end-of-life management. Consequently, the environmental benefit of a modified natural-fibre composite depends not only on the renewable origin of the reinforcement but also on matrix selection, processing route, service-life extension and the adopted end-of-life scenario. Recent comparative life-cycle assessment (LCA) studies demonstrate that the environmental performance of natural-fibre composites depends strongly on material composition, matrix selection, fibre treatment and manufacturing route. Authors in [121] compared bio-based composite systems containing flax fibres and bio-based polymer matrices with conventional glass-fibre-reinforced alternatives and demonstrated that substitution of synthetic reinforcement and fossil-based constituents can reduce selected environmental impacts. However, the magnitude of these benefits depends strongly on the specific fibre–matrix system, fibre-treatment route and manufacturing process considered in the life-cycle assessment [121]. This trade-off becomes particularly important when additional fibre treatments are introduced to improve durability. Recent LCA-oriented studies further demonstrate that improvements in mechanical performance and moisture resistance achieved through fibre modification may be accompanied by a substantially higher environmental burden associated with chemical treatment [105]. These results demonstrate that the treatment providing the greatest short-term technical improvement does not necessarily represent the environmentally preferable solution over the complete material life cycle. A similar durability–environmental trade-off was identified in [122], which showed that fibre surface treatment constituted the most environmentally impactful stage in the investigated PLA-based natural-fibre biocomposite systems. At the same time, silane fibre treatment and the use of a reactive chain extender helped limit the deterioration associated with hygrothermal ageing and subsequent mechanical reprocessing, although their effectiveness depended on the composite formulation and treatment combination [122]. These findings highlight an important optimisation problem: additional modification may improve durability and property retention during subsequent processing, but the environmental burden associated with the treatment itself must be balanced against the resulting extension of functional service life and the potential for material recovery. The recent literature therefore shows that durability enhancement and environmental sustainability cannot be optimised independently. Chemical treatments, coupling agents and multifunctional coatings may improve moisture resistance, interfacial stability and biological durability, but they may also increase processing energy demand, chemical consumption and end-of-life complexity. Conversely, lower-impact treatments may provide smaller short-term property improvements while offering better recyclability or lower embodied environmental burden. Future material optimisation should therefore consider durability, environmental impact and end-of-life performance as coupled design objectives rather than independent criteria.

### Recyclability, Biodegradation and End-of-Life Implications

End-of-life performance represents an additional challenge because the same modifications that improve resistance to biodeterioration during service may adversely affect recyclability or biodegradability after service failure. This creates an inherent durability–circularity trade-off. Increasing resistance to moisture, microbial attack and enzymatic degradation is desirable during the service life of a building component, but persistent hydrophobic treatments, cross-linked coatings or antimicrobial additives may subsequently hinder biological decomposition, material separation or recycling. Consequently, anti-biodeterioration strategies should be evaluated not only according to their protective efficiency during use but also according to their influence on available end-of-life pathways.

Impacts of various fibre treatment and protection methods on material circularity are outlined below:

1. Alkali treatment and silane: Fibre surface treatments have different implications for circularity. Alkali treatment primarily modifies the lignocellulosic fibre surface by removing non-cellulosic constituents and altering surface morphology and therefore does not introduce a persistent additional material phase. Silane coupling, in contrast, creates more stable chemical interactions at the fibre–matrix interface and can improve resistance to moisture and repeated processing. Although such modification may enhance property retention during recycling, stronger interfacial bonding and additional chemical functionality may also influence fibre separation and subsequent biodegradation. The overall end-of-life effect therefore depends strongly on matrix type and the selected recycling route [122].
2. Hydrophobic coatings: Hydrophobic surface treatments present a different trade-off. By reducing water penetration, they may substantially delay moisture-induced degradation during service; however, persistent coatings can complicate mechanical recycling and may reduce the accessibility of lignocellulosic components to microorganisms during biological end-of-life treatment. Multilayer coatings are particularly problematic when chemically incompatible materials must be separated before recycling. Therefore, coating durability should ideally be accompanied by design-for-disassembly or selective-removal strategies.
3. Chitosan: Bio-based protective agents such as chitosan may offer advantages from a circular-economy perspective because they originate from renewable resources and are inherently biodegradable under appropriate conditions. Nevertheless, biodegradability of the protective agent alone does not guarantee biodegradability of the complete composite. Cross-linking, chemical functionalisation and combination with non-biodegradable polymer matrices may substantially alter end-of-life behaviour. Chitosan-based treatments should therefore be evaluated at the level of the complete material system rather than being classified as environmentally benign solely on the basis of feedstock origin.
4. TiO_2_ and ZnO: Nanoparticle-based antimicrobial systems introduce additional end-of-life concerns. TiO_2_ and ZnO are persistent inorganic phases that do not biodegrade together with lignocellulosic reinforcement or biodegradable polymer matrices. During mechanical recycling, weathering, fragmentation or biological degradation of the surrounding matrix, nanoparticles may remain in the recycled fraction or be released into the environment. Their long-term fate depends on particle size, surface functionalisation, matrix binding and the recycling or disposal process. Consequently, antimicrobial efficiency during service should be balanced against potential nanoparticle release, ecotoxicological effects and contamination of secondary material streams.
5. Hybrid systems: Hybrid protection systems present the greatest optimisation challenge because they combine several material modifications and may consequently provide superior durability while increasing compositional complexity. Systems incorporating fibre pretreatment, coupling agents, hydrophobic coatings and antimicrobial nanoparticles can act at multiple stages of the biodeterioration pathway, but the resulting multi-material architecture may be more difficult to recycle or biodegrade selectively. This illustrates a potential conflict between maximising service-life durability and maintaining simple, circular end-of-life pathways.

The principal end-of-life implications of the anti-biodeterioration strategies discussed in this review are compared in Table 10.

As shown in Table 10, the circularity implications of durability-enhancing treatments differ substantially according to the modification mechanism and the composition of the complete composite. Alkali treatment introduces no persistent additional material phase, whereas coupling agents, coatings and nanoparticle-based systems may increase the chemical and compositional complexity of the resulting composite [95,123,124]. Chitosan-based treatments may offer advantages because of the renewable and biodegradable nature of the biopolymer itself; however, this advantage cannot be extrapolated automatically to the complete composite, particularly when cross-linkers, synthetic matrices or inorganic nanoparticles are present [20,125]. The most significant knowledge gap concerns multifunctional and nanoparticle-modified systems, for which integrated long-term studies combining service ageing, recycling, biodegradation and additive-release assessment remain scarce [126,127]. Importantly, greater resistance to biodegradation during service is not necessarily compatible with rapid biodegradation after disposal. This apparent contradiction highlights the need to distinguish between controlled biological durability during the service phase and desirable end-of-life degradability. An optimal circular design should maintain resistance to moisture and microbial attack throughout the intended service life while enabling appropriate end-of-life pathways, including recycling, component separation or, where technically applicable, controlled biodegradation [20,124]. Future studies should therefore evaluate anti-biodeterioration treatments within a full life-cycle framework that includes not only initial antifungal efficacy and property retention but also repeated reprocessing, recyclate quality, disassembly potential, biodegradation kinetics and the fate of functional additives [122,126,127]. Such an approach would allow the environmental benefits associated with service-life extension to be balanced against additional processing burdens and end-of-life constraints.

The comparison demonstrates that no modification strategy can currently be identified as universally optimal from both durability and circularity perspectives. The environmental value of a treatment depends on whether the functional or service-life benefits achieved justify the additional environmental burdens and end-of-life complexity introduced by the modification [105,121,122]. This balance is particularly important for construction products, where service-life extension can substantially influence life-cycle environmental performance; however, the magnitude of this benefit depends on the material system, replacement scenario and assumptions adopted in the life-cycle assessment. Conversely, highly effective protective systems may become environmentally less favourable if they hinder material recovery or introduce persistent functional components into recycling or waste-treatment streams [126,127].

Future research should therefore adopt a safe-and-circular-by-design perspective, in which anti-biodeterioration efficiency, expected service-life extension, recyclability, biodegradability and potential release of functional additives are assessed as interconnected design criteria. Comparative studies should report not only initial antifungal efficacy and retained mechanical properties but also performance after repeated recycling, recyclate quality, disassembly potential, additive release and end-of-life environmental impacts [122,126,127]. Such an approach would enable optimisation of biological durability while reducing the risk of shifting environmental burdens from the use phase to recycling and end-of-life stages.

Field-scale evidence remains substantially more limited for polymer-matrix natural fibre composites than for related bio-based construction materials, such as timber, wood-based products and plant-fibre insulation. Most durability data for NFRPCs are derived from accelerated laboratory protocols involving water immersion, constant high relative humidity, controlled fungal inoculation or short-term hygrothermal ageing. Although these methods are essential for identifying individual degradation mechanisms, they do not reproduce the full combination of seasonal temperature and humidity fluctuations, intermittent wetting and drying, solar radiation, surface contamination and naturally occurring microbial communities encountered under actual service conditions. The recent literature therefore continues to identify the scarcity of long-term field data as a major limitation in the durability and service-life assessment of natural-fibre composite and related bio-based construction systems [40,60,87].

The distinction between laboratory and field exposure is particularly important for biodeterioration. Continuous high humidity and controlled inoculation may promote rapid fungal development, whereas real building components undergo alternating wetting and drying periods that can interrupt biological activity while simultaneously inducing repeated fibre swelling, shrinkage and interfacial damage. Moreover, laboratory inoculation with individual fungal species does not reproduce the succession and competition of naturally occurring microbial communities. Consequently, degradation rates obtained from accelerated tests should not be interpreted directly as service-life conversion factors unless they are validated against natural-weathering or in-service data.

**Figure 12:**
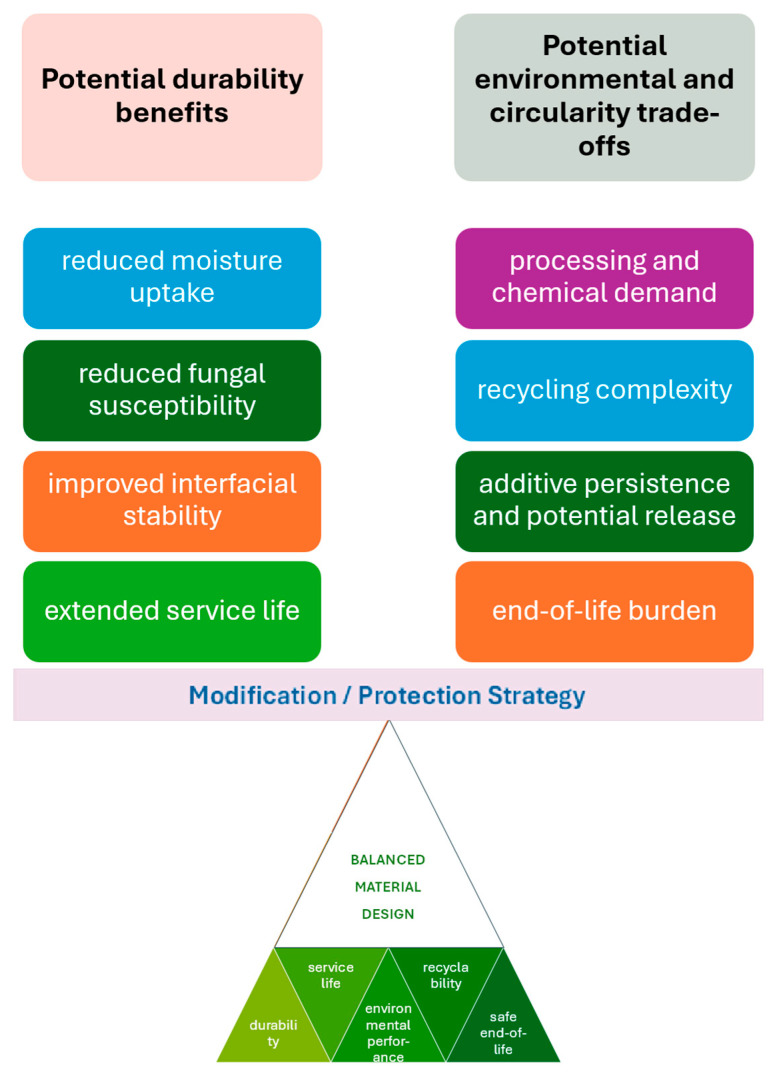
Conceptual durability–circularity trade-off associated with protection and modification strategies for natural fibre-reinforced construction biocomposites. Potential durability benefits include reduced moisture uptake and fungal susceptibility, improved interfacial stability and extended service life, whereas potential environmental and circularity trade-offs may arise from processing and chemical demand, additive persistence, recycling complexity, material release and end-of-life management. The relative balance of these effects is treatment- and system-dependent; therefore, the figure represents a conceptual decision framework rather than a quantitative ranking of individual protection strategies. Prepared by the author based on the reviewed literature.

Available field evidence from related bio-based building materials nevertheless demonstrates that component orientation, construction detailing, exposure to driving rain, ventilation and drying potential strongly influence biological durability under natural conditions. These results cannot be transferred quantitatively to polymer biocomposites because the polymer matrix modifies water transport, fibre accessibility and microbial colonisation; however, they demonstrate that exposure conditions and construction design may be as important as intrinsic material resistance in determining long-term performance. Future studies should therefore combine accelerated laboratory ageing with natural outdoor exposure and in-service monitoring of the same material systems, using parallel measurements of moisture, microbial colonisation, microstructural changes and residual mechanical and thermal properties. Improving resistance to moisture and biodeterioration does not necessarily result in an unambiguous improvement in the overall environmental performance of a biocomposite. Protection and modification strategies may extend service life by reducing moisture uptake, limiting fungal susceptibility and preserving fibre–matrix interfacial integrity; however, additional processing, chemical treatments, persistent additives or increasingly complex multi-component systems may adversely affect recyclability and end-of-life management. The magnitude and relevance of these trade-offs depend on the treatment chemistry, material formulation, intended application and end-of-life scenario. Consequently, protection strategies should be evaluated not only in terms of durability improvement but also from a broader life-cycle and circularity perspective, as conceptualised in Figure 12.

The optimal protection strategy should therefore not be defined by maximum resistance to biodeterioration alone. Instead, material design should balance the durability required for the intended exposure conditions with environmental performance, recyclability and safe end-of-life management. Importantly, individual protection strategies cannot currently be ranked universally within this trade-off because comparative life-cycle and long-term durability datasets remain limited and strongly dependent on material formulation, processing route and application conditions.

## 8. Conclusions and Future Outlook

Natural fibre-reinforced polymer biocomposites offer a potentially lower-impact alternative for selected construction applications, but their service performance cannot be judged from mechanical properties or fungal resistance alone. The key durability problem is coupled: moisture uptake changes fibre dimensions, weakens the fibre–matrix interphase, modifies the pore network and enables microbial activity. These processes act together, so visible colonisation represents only one component of the overall degradation process.

The strongest evidence reviewed in this paper concerns humid exposure, water absorption and the resulting loss of mechanical performance. Across the quantitative datasets reviewed, tensile-strength losses under moisture and hygrothermal exposure generally ranged from several percent to approximately 40%, while substantially larger reductions were reported for selected mechanical indicators. Evidence is less consistent when biological attack, cyclic wetting and drying, thermal insulation performance and microstructural damage are evaluated separately. Reported deterioration should therefore be interpreted in relation to fibre type, matrix chemistry, fungal species, exposure duration and moisture history; without these parameters, comparisons between studies remain uncertain.

For construction applications, ISO 846 and related laboratory protocols provide useful screening information, but they are not sufficient as stand-alone durability tests for porous and hygroscopic biocomposites. Future protocols should integrate the assessment of fungal development with measurements of moisture content, water activity, drying behaviour, residual mechanical properties, thermal conductivity and microstructural changes. This shift from isolated fungal screening to coupled durability evaluation is essential for materials intended for building envelopes and other long-service-life applications.

Protection strategies should also be assessed within this coupled framework. Alkali and silane treatments, hydrophobic coatings, bio-based protective systems and antimicrobial or photocatalytic nanoparticles can improve selected aspects of durability, but their effectiveness depends on their ability to control moisture ingress, preserve interfacial integrity and maintain functionality under cyclic hygrothermal exposure. Combined protection systems matched to the fibre, matrix and intended exposure class appear particularly promising because they can target multiple stages of the degradation pathway. However, their long-term superiority over simpler single-treatment strategies has not yet been demonstrated consistently under realistic service conditions.

Durability enhancement should not be considered independently of environmental and end-of-life performance. Treatments that reduce moisture uptake or microbial colonisation may introduce additional processing burdens, persistent additives or recycling constraints. Their environmental value therefore depends on whether the resulting service-life extension compensates for production impacts and end-of-life complexity. Future material development should consequently integrate durability, life-cycle assessment, recyclability and controlled end-of-life pathways within a safe-and-circular-by-design framework.

The main limitation of current research is not the absence of degradation data, but the weak connection between biological exposure, moisture history and residual engineering performance. A composite showing limited visible fungal growth may still accumulate moisture, lose interfacial integrity or exhibit increased thermal conductivity after service-like exposure. Future studies should therefore integrate controlled hygrothermal and biological exposure with complementary diagnostic techniques such as acoustic emission, X-ray micro-computed tomography and microscopy, and use the resulting longitudinal datasets to develop and validate coupled service-life models. AI-assisted prediction and digital-twin frameworks may support this process, but their reliability will depend on sufficiently large, representative and long-term datasets linking environmental exposure with biological, microstructural and functional deterioration. A critical next step is validation through long-term outdoor exposure and in-service monitoring because accelerated laboratory ageing cannot yet be translated directly into reliable service-life estimates for construction components.

## Figures and Tables

**Table 1:** Characteristics of natural fibres used in construction biocomposites (Prepared by the author based on the literature [5,8,9,31]).

Fibre	Cellulose [%]	Hemicellulose [%]	Lignin [%]	Density [g/cm^3^]
Hemp	55–72	8–19	2–5	1.48
Flax	65–85	10–20	2–5	1.40–1.50
Jute	60–71	13–20	12–14	1.30–1.46
Kenaf	45–57	21–23	8–13	1.20–1.45
Straw	35–45	20–30	15–20	0.90–1.30

**Table 2:** Representative construction applications of natural fibre-reinforced biocomposites.

Construction Application	Typical Natural Fibre	Matrix/Binder	Key Functional Properties	Representative Literature
Structural and semi-structural composite panels	Flax	Epoxy	High specific strength, stiffness, low density	[9,34]
Fibre-reinforced polymer composites for building components	Flax, hemp, jute	PP, PE, polyester	Improved mechanical performance after interface modification	[33,35]
Thermal insulation materials	Hemp shiv	Lime binder (hemp-lime)	Low thermal conductivity, moisture buffering, low embodied carbon	[36]
Lightweight bio-based insulation	Flax, hemp	Various bio-based binders	Low thermal conductivity and good hygrothermal performance	[37]
Natural fibre polymer composites for building applications	Flax, hemp, kenaf	Thermoplastics and thermosets	Balance between mechanical properties and environmental performance	[7,35]

**Table 3:** Application-oriented classification of natural fibre-based construction biocomposites and associated biodeterioration risks. Classification developed by the author based on the reviewed literature.

Functional Category	Representative Construction Applications	Typical Exposure/Service Conditions	Critical Performance Criteria	Main Biodeterioration-Related Mechanisms	Principal Engineering Consequences
Load-bearing	Structural sandwich panels, fibre-reinforced profiles, decking systems	Sustained/cyclic loading; possible moisture fluctuations	Strength, stiffness, fatigue resistance, interfacial integrity	Moisture ingress, fibre swelling, fungal degradation of lignocellulosic reinforcement, fibre–matrix interfacial damage	Loss of stiffness and strength; reduced structural reliability
Semi-load-bearing	Façade and cladding panels, partitions, ceiling elements, door components, prefabricated enclosure panels	Cyclic RH and temperature; condensation; wetting–drying; outdoor exposure in façade applications	Dimensional stability, surface integrity, moisture resistance, retained mechanical performance	Moisture cycling, swelling/shrinkage, interfacial degradation, surface fungal colonisation	Warping, cracking, delamination, surface deterioration and loss of serviceability
Non-load-bearing/insulating	Insulation boards, lightweight infill panels, porous bio-based components in wall, roof and floor assemblies	High RH; condensation; prolonged local moisture retention; limited drying	Low thermal conductivity, moisture management, dimensional stability, biological resistance	Moisture accumulation enabling fungal colonisation; microbial degradation; changes in pore structure and moisture-transport behaviour	Moisture-related increase in λ, reduced insulation efficiency, biodeterioration-related material damage and potential indoor microbial exposure

**Table 4:** Major microorganisms responsible for the degradation of construction biocomposites.

Microorganism	Moisture Requirements	Main Degradation Mechanism	Typical Deterioration Effect	Source
*Aspergillus niger*	Moderate–high humidity	Cellulolytic enzymes	Surface colonisation, reduction in strength	[17]
*Penicillium chrysogenum*	High humidity	Hydrolysis of polysaccharides	Loss of cohesion	[17]
*Cladosporium cladosporioides*	High relative humidity	Surface colonisation	Surface staining, biofilm formation	[57]
*Trichoderma viride*	High moisture availability	Intensive cellulolytic activity	Rapid cellulose degradation	[58]
*Chaetomium globosum*	Long-term moisture exposure	Cell wall degradation	Deep structural deterioration	[59]

**Table 5:** Changes in mechanical and physical properties of selected biocomposites following moisture exposure or biodeterioration.

Author	Material	Exposure Condition	Main Deterioration Effects
[15]	Hemp fibre-reinforced unsaturated polyester composite	Immersion in water (25 °C and 100 °C)	An increase in fibre content increased moisture absorption. As absorption increased, a decrease in tensile and flexural strength and degradation of the fibre–matrix interphase were observed
[17]	Flax and hemp mats, as well as flax/PP and hemp/PP composites	28-day exposure to *Chaetomium globosum* under high-humidity conditions	The fungus caused a loss of dry weight in the material, a decrease in the elastic modulus, and the development of microstructural damage observed via SEM
[61]	Flax fibre-reinforced PLA composite	Hydrothermal ageing	Decrease in stiffness and mechanical properties
[36]	natural fibre composites	Review of environmental studies	Moisture leads to fibre swelling, weakened interfacial adhesion, increased porosity, and deterioration of the composites’ mechanical properties
[63]	Non-woven flax/PLA composite	98% RH and water immersion	Tangent modulus decreased by 23.0% at 98% RH and 33.8% after immersion; ultimate strength decreased by 26.7% and 37.4%, respectively
[64]	Woven flax/PLA composite	40 °C, 100% RH, up to 42 days	Stiffness decreased by >50% within 24 h and by ~72% after prolonged ageing; tensile strength decreased by ~31% after 42 days

**Table 6:** Quantitative comparison of mechanical degradation reported for selected natural fibre-reinforced polymer composites under moisture, hygrothermal and biological exposure.

Fibre/Matrix	Exposure	Conditions	Mechanical Response	Author
Flax/PLA, non-woven	High-humidity ageing	98% RH	Tangent modulus: −23.0%; ultimate strength: −26.7%	[63]
Flax/PLA, non-woven	Water ageing	Immersion	Tangent modulus: −33.8%; ultimate strength: −37.4%	[63]
Flax/PLA, woven	Hygrothermal ageing	40 °C, 100% RH, 42 d	Stiffness: −72%; strength: −31%	[64]
Flax–glass hybrid/epoxy	Environmental/hygrothermal exposure	Depending on exposure regime	Tensile-strength retention: 65.4–88.0%	[67]
Flax–glass hybrid/polyurethane	Environmental/hygrothermal exposure	Depending on exposure regime	Tensile-strength retention: 80.7–94.1%	[67]
Hemp fibre/polymer	Combined environmental ageing	UV–thermal–moisture exposure	Tensile strength: −9.57 to −22.12%; impact strength: −38.68 to −46.03%	[65]
Hemp- and flax-based polymer composites	Fungal exposure	Moisture + cellulolytic fungi	Significant deterioration of fibre integrity and mechanical performance; magnitude dependent on material architecture	[17]
Wood–polymer composites	Weathering followed by biological exposure	Natural pre-weathering + fungal durability testing	Biological resistance dependent on previous weathering history; Not quantitatively reported	[69]
Wood fibre/PLA-based hybrid composites	Fungal decay	Fungal exposure	Susceptibility increased with increasing lignocellulosic reinforcement content; Not quantitatively reported	[70]

**Table 7:** Quantitative evidence linking fungal contamination, indoor exposure indicators and deterioration of bio-based building materials.

Indicator	Material/Environment	Quantitative Result	Demonstrated Relationship	Direct Correlation with Mechanical/Thermal Degradation
Airborne fungi	Moisture/flood-affected buildings	Up to ~2.4 × 10^3^ CFU/m^3^	Moisture damage associated with elevated fungal aerosol	No
Dust-borne fungi	Moisture/flood-affected buildings	Up to ~2.5 × 10^5^ CFU/g	Dust acts as microbial reservoir	No
Total mould/VOC exposure	Residential buildings, 159 adults	Total mould associated with oculo-nasal symptoms (*p* = 0.038)	Quantitative association with occupant symptoms	No
Selected VOCs	Residential indoor air	3-methylfuran: *p* = 0.003; 2-hexanone: *p* = 0.037; 2-heptanone: *p* = 0.018; 1-octen-3-ol: *p* = 0.001	Significant associations with oculo-nasal symptoms	No
Fungal-associated VOC profile	Indoor fungi vs common building-material emissions	Random forest accuracy: 96.2%	Effective discrimination of fungal contamination	No
Mechanical degradation	Natural-fibre composites	Strength/stiffness losses quantified in Section 4.1	Moisture/fungal exposure → property deterioration	Not simultaneously correlated with IAQ indicators
Thermal degradation	Bio-based building materials	Δλ quantified in Section 4.2	Moisture → increased λ	Direct fungal burden–Δλ relationship not established

**Table 8:** Comparative assessment of hybrid and multifunctional protection strategies relevant to the biodeterioration resistance of natural fibre-based construction biocomposites.

Hybrid Strategy	Evidence Level	Primary Mechanisms	Potential Synergistic Advantage	Main Limitations	Relevant Building Application
Alkali + silane [90,95]	Direct evidence for combined fibre modification and improved interfacial properties; limited direct biodeterioration evidence	Fibre surface cleaning/fibrillation + chemical coupling	Improved fibre–matrix adhesion and reduced interfacial moisture susceptibility	Excessive alkalisation may damage fibres; effectiveness depends on treatment conditions	Structural and semi-structural polymer composites exposed to cyclic humidity
Silane-based interface modification in hybrid natural-fibre composites [96]	Direct composite-level evidence for interface/property modification; no direct antifungal validation	Silane-mediated fibre–matrix coupling and interfacial modification	Improved interfacial compatibility in hybrid fibre–filler polymer systems	No direct antifungal validation; durability under cyclic moisture exposure requires further assessment	Façades, cladding and semi-exposed components
Chitosan + TiO_2_ [97,98,99]	Direct antifungal evidence in coating systems and related bio-based substrates; no direct long-term validation identified for construction-grade NFRPCs	Antimicrobial activity of chitosan + TiO_2_-mediated photocatalytic activity	Complementary passive and active antimicrobial mechanisms	Chitosan moisture sensitivity; irradiation dependence; transferability to construction composites requires validation	Interior/coated panels and potentially semi-exposed surfaces
ZnO-functionalised natural-fibre systems [100]	Experimental evidence reported across natural-fibre composite systems; limited long-term fungal and field-scale validation	Functional modification + ROS/Zn^2+^-related antimicrobial activity	Potential combination of moisture resistance and antimicrobial functionality	Nanoparticle release, ecotoxicity and limited field validation	Moisture-prone enclosure and surface applications
TiO_2_-functionalised natural-fibre systems [101,102,103,104]	Direct experimental evidence for TiO_2_-modified flax fibre/polymer composites [101,102] and for antifungal or antimicrobial activity of TiO_2_ in related construction-material systems [103,104]; limited direct long-term fungal durability evidence in TiO_2_-modified NFRPCs (Natural fibre-reinforced polymer composites).	ibre/interface and moisture-related modification + TiO_2_-mediated photocatalytic ROS generation	Combination of improved fibre–matrix/moisture-related performance with potential photocatalytic antimicrobial functionality	Dependence of photocatalytic activity on irradiation, nanoparticle dispersion and accessibility; limited long-term fungal and field-scale validation	Light-exposed panels, façades and coated composite surfaces
Integrated multistage fibre/interface modification with antimicrobial functionality [90,97,98,99,100] *	Conceptual/emerging strategy derived from complementary modification approaches; limited direct validation of the complete integrated system	Fibre modification + interface improvement + antimicrobial functionality	Potential multilevel protection of fibre, interface and exposed surface	Processing complexity, cost, compatibility, recyclability and lack of long-term field data	Potential future high-durability structural and semi-structural applications
Propolis + silane [94]	Direct experimental evidence in lignocellulosic–polymer composites	Silane-based surface modification + bioactive antifungal action of propolis	Combination of interface/surface modification with antifungal protection	Long-term durability, leaching and performance under cyclic environmental exposure require further validation	Panels, WPC-type components and moisture-exposed composite surfaces

* Note: Evidence levels indicate the degree of experimental validation reported for each strategy in natural fibre/polymer composites or closely related bio-based material systems. For emerging integrated strategies, cited references may support individual functional components rather than direct experimental validation of the complete treatment combination. Potential building applications are inferred from the functional characteristics of the protection strategies and the intended use of the corresponding material systems; they should not be interpreted as evidence of field-scale validation.

**Table 9:** Current state of knowledge, major research gaps and future research priorities for the biological durability of natural fibre-reinforced polymer biocomposites used in construction. Prepared by the author based on the literature [6,12,13,14,16,17,46].

Research Area	Current State of Knowledge	Main Research Gap	Future Research Priority
Biodeterioration	Numerous laboratory studies	Lack of in situ investigations	Long-term monitoring of real buildings
Mechanical properties	Well characterised	Lack of long-term durability studies	Service-life assessment (>5 years)
Thermal performance	Thermal conductivity (λ) well documented	Lack of coupled hygrothermal–biological studies	Monitoring of λ during biodeterioration
Microstructure	SEM, FTIR and MIP widely applied	Limited correlation with functional properties	Integrated SEM–µCT–MIP analysis
Protective coatings	Promising laboratory performance	Lack of field validation	Validation under real service conditions
Nanomaterials	Promising antimicrobial performance	Limited information on long-term durability and environmental impact	Long-term durability assessment and life cycle assessment (LCA)
Service-life prediction	Empirical and mechanistic models available for selected degradation processes; emerging data-driven approaches	Lack of validated coupled models linking moisture, fungal activity, microstructural damage and functional-property loss	Validation of coupled models using long-term field data; integration with AI-assisted prediction and digital-twin frameworks
Standardisation	Existing standards developed mainly for synthetic polymers	No dedicated standards for bio-based composites	Development of harmonised testing protocols

**Table 10:** End-of-life implications of anti-biodeterioration modification strategies for natural fibre-based polymer biocomposites.

Modification Strategy	Durability Benefit During Service	Recyclability Implications	Biodegradation/End-of-Life Implications	Evidence Status	Main Circular-Economy Concern	Representative Literature
Alkali treatment	Removes surface impurities and part of non-cellulosic constituents; may improve fibre–matrix adhesion	Does not introduce a separate persistent material phase; recyclability remains primarily controlled by matrix type and fibre damage during reprocessing	Lignocellulosic fibre remains biodegradable in principle, although treatment severity can alter fibre structure	Direct evidence for fibre modification; limited direct EoL comparisons	Excessive treatment may damage fibres; repeated processing shortens fibres and can reduce performance	[95,123,124]
Silane coupling	Improves fibre–matrix interface, moisture resistance and retention of properties	Stronger interface may favour property retention during reprocessing, but the effect on fibre recovery/separation depends on matrix and recycling route	Surface chemical modification may influence degradation kinetics; insufficient evidence for a universal effect on biodegradation of complete composites	Direct durability evidence; limited EoL evidence	Trade-off between durable interface and ease of material separation/recovery	[95,123,124]
Hydrophobic treatments/coatings	Reduce water ingress and moisture-driven deterioration	Additional coating layers may complicate recycling when chemically incompatible with the substrate or matrix	Persistent barriers can potentially reduce water/microbial access to the underlying bio-based phase; effect is coating-specific	System-dependent; direct long-term EoL evidence limited	Multimaterial complexity, coating removal and compatibility with recycling streams	[95,124]
Chitosan-based protection	Provides bio-based antimicrobial/fungistatic functionality and may reduce microbial colonisation of susceptible surfaces	Recyclability depends on the matrix and overall composite formulation; cross-linkers and additional functional components may affect available recycling routes	Chitosan is biodegradable, but biodegradability of the complete modified composite depends on matrix type, cross-linking and other additives	Antimicrobial functionality and biodegradability of chitosan are well established; direct EoL evidence for chitosan-modified construction NFRPCs remains limited	Renewable and biodegradable origin of chitosan does not ensure biodegradability or recyclability of the complete modified composite	[20,125]
TiO_2_/ZnO nanoparticle modification	Provides antimicrobial/photocatalytic functionality and may improve selected moisture/functional properties	Inorganic nanoparticles may remain associated with recycled fractions; behaviour depends on matrix binding and recycling technology	TiO_2_ and ZnO are inorganic and do not biodegrade with the organic composite fraction; matrix degradation may alter their environmental availability	Functional performance established; EoL fate in NFRPCs insufficiently characterised	Potential nanoparticle release, accumulation in secondary streams and ecotoxicological uncertainty	[100,101,102,103,104,126,127]
Hybrid/multifunctional systems	Simultaneously target moisture ingress, interface degradation and microbial colonisation	Multiple treatments and material phases can increase recycling complexity and hinder clean material separation	Overall biodegradability is determined by the least degradable/persistent components and their interactions	Emerging; very limited direct EoL validation	Potential conflict between maximum service-life durability and circular end-of-life pathways	[20,124]

## Data Availability

No new data were created or analysed in this study. Data sharing is not applicable to this article.

## Abbreviations

The following abbreviations are used in this manuscript:

AI	Artificial Intelligence
ASTM	ASTM International (formerly American Society for Testing and Materials)
CNN	Convolutional Neural Network
DT	Digital Twin
EN	European Standard
ISO	International Organisation for Standardisation
IAQ	Indoor Air Quality
LCA	Life-Cycle Assessment
ML	Machine Learning
MVOCs	Microbially Produced Volatile Organic Compounds
NFRPCs	Natural Fibre-Reinforced Polymer Composites
PLA	Polylactic Acid
PP	Polypropylene
PVC	Poly(vinyl chloride)
SBS	Sick Building Syndrome
SEM	Scanning Electron Microscopy
TiO_2_	Titanium Dioxide
UV	Ultraviolet
VOCs	Volatile Organic Compounds
WPCs	Wood–Plastic Composites
ZnO	Zinc Oxide
